# Small RNAs positively and negatively control transcription elongation through modulation of Rho utilization site accessibility

**DOI:** 10.1128/mbio.02921-25

**Published:** 2025-10-31

**Authors:** Kristen R. Farley, Andrew K. Buechler, Colleen M. Bianco, Carin K. Vanderpool

**Affiliations:** 1 Department of Microbiology, University of Illinois Urbana-Champaign 14589, Urbana, Illinois, USA; 2 Carl R. Woese Institute of Genomic Biology, University of Illinois Urbana-Champaign 14589, Urbana, Illinois, USA; University of California, Berkeley, Berkeley, California, USA

**Keywords:** Hfq, RNase E, termination, small RNA

## Abstract

Bacteria use a multi-layered regulatory strategy to precisely and rapidly tune gene expression in response to environmental cues. Small RNAs (sRNAs) form an important layer of gene expression control and most act post-transcriptionally to control translation and stability of mRNAs. We have shown that at least five different sRNAs (RydC, CpxQ, ArrS, GcvB, and OxyS) in *Escherichia coli* regulate the cyclopropane fatty acid synthase (*cfa*) mRNA. These sRNAs bind at different sites in the long 5′ untranslated region (UTR) of *cfa* mRNA, and previous work suggested that they modulate RNase E-dependent mRNA turnover. Rho-dependent transcription termination in the *cfa* 5′' UTR was demonstrated, leading us to hypothesize that the sRNAs might also regulate *cfa* transcription elongation. In this study, we find that a pyrimidine-rich region flanked by sRNA binding sites in the *cfa* 5′ UTR is required for premature Rho-dependent termination. We discovered that sRNA-dependent regulation of *cfa* depends on Rho, and the activating sRNA RydC has only a minor effect on RNase E-mediated turnover of *cfa* mRNA. A stem-loop structure in the *cfa* 5′ UTR sequesters the pyrimidine-rich region required for Rho-dependent termination. The repressing sRNA CpxQ binds to the 5′ portion of the stem and increases Rho-dependent termination, whereas the activating sRNA RydC binds downstream of the stem and decreases termination. These results reveal the versatile mechanisms sRNAs use to regulate target gene expression at transcriptional and post-transcriptional levels and demonstrate that regulation by sRNAs in long UTRs can involve modulation of transcription elongation.

**IMPORTANCE:**

Bacteria respond to stress by rapidly regulating gene expression. Regulation can occur through the control of messenger RNA (mRNA) production (transcription elongation), stability of mRNAs, or translation of mRNAs. Bacteria can use small RNAs (sRNAs) to regulate gene expression at each of these steps, but we often do not understand how this works at a molecular level. In this study, we find that sRNAs in *Escherichia coli* regulate gene expression at the level of transcription elongation by promoting or inhibiting transcription termination by a protein called Rho. These results help us understand new molecular mechanisms of gene expression regulation in bacteria.

## INTRODUCTION

Small RNA (sRNA) regulators in bacteria, archaea, and eukaryotes promote rapid changes in gene expression and subsequent changes in cell physiology or behavior (1). Many sRNAs exert their effects by short semi-complementary base pairing interactions with target messenger RNAs (mRNAs) (2, 3). In *Escherichia coli* and related bacteria, sRNAs require the RNA chaperone Hfq for stability and mRNA target regulation (4). Many Hfq-dependent sRNAs base pair near the ribosome binding site (RBS) on mRNAs to modulate translation. Some sRNAs directly modulate mRNA stability by blocking ribonuclease E (RNase E) cleavage sites (4, 5). Although bacterial sRNAs are best known as post-transcriptional regulators, some sRNAs modulate transcription elongation through interactions in long 5′ untranslated regions (UTRs) of mRNAs (6, 7). About 25% of *E. coli* mRNAs possess 5′ UTRs longer than 80 nt, and the levels of approximately half of these long-UTR mRNAs are altered by bicyclomycin, an inhibitor of transcription termination factor Rho (7). These observations suggest that Rho-dependent termination events in long 5′ UTRs may be common and that more sRNAs could regulate gene expression by modulating transcription elongation.

Rho is an essential transcription termination factor in Proteobacteria (8). It binds to nascent transcripts at sequences called Rho utilization (*rut*) sites and hydrolyzes ATP to translocate toward the transcription elongation complex. Rho destabilizes the complex, leading to transcription termination (8). Active translation shields mRNAs from Rho-dependent transcription termination within coding sequences, but uncoupling of transcription and translation can leave nascent mRNAs susceptible to premature Rho-dependent termination (8). While it is evident how sRNA-mediated translational repression renders target mRNAs susceptible to both RNases and Rho, it has been unclear how sRNA regulators that do not directly affect translation modulate transcription elongation or mRNA stability (6). Nevertheless, there are several examples of such translation-independent regulation. The sRNAs DsrA, ArcZ, and RprA prevent Rho-dependent termination within the *rpoS* mRNA 5′ UTR (7) by a mechanism proposed to involve disruption of Rho binding or translocation. The sRNA SraL represses termination within the *rho* mRNA, which is autoregulated (9, 10). SraL is thought to induce *rho* mRNA structural changes to make a *rut* site inaccessible (10).

The *cfa* mRNA in *E. coli* is regulated positively and negatively by sRNAs that base pair at different sites within a 212-nt 5′ UTR (11, 12). The *cfa* gene encodes cyclopropane fatty acid (CFA) synthase that installs cyclopropane rings in unsaturated membrane phospholipids (13). Under acidic and oxidative stress conditions, CFAs are thought to promote survival by stabilizing the membrane and reducing permeability (13, 14). The sRNAs RydC, ArrS, and OxyS are activators of *cfa* (11, 12) while CpxQ (15) and GcvB repress *cfa* (11). Previous studies suggested that these sRNAs regulate *cfa* by modulating RNase E-mediated *cfa* mRNA decay (11, 12, 16). However, there is evidence for Rho-dependent transcription termination in the 5′ region of the *cfa* coding sequence (CDS) (17), prompting us to investigate whether sRNAs regulate *cfa* through modulating Rho-dependent termination.

In this study, we found that Rho limits transcription of a long 5′ UTR isoform of *cfa* mRNA. The sRNA binding sites within the *cfa* 5′ UTR flank a pyrimidine-rich region that has hallmarks of a *rut* site, and this region is critical for Rho-dependent *cfa* regulation. The activating sRNAs ArrS and RydC, as well as the repressing sRNAs CpxQ and GcvB, all require Rho to carry out the regulation of *cfa*. Our data suggest that sRNAs primarily act via modulation of Rho-dependent termination to regulate *cfa*. This work reveals that some sRNAs regulate their mRNA targets in a translation-independent manner at the level of transcription elongation.

## RESULTS

### Rho regulates transcription of the long *cfa* mRNA isoform

**Fig 1:**
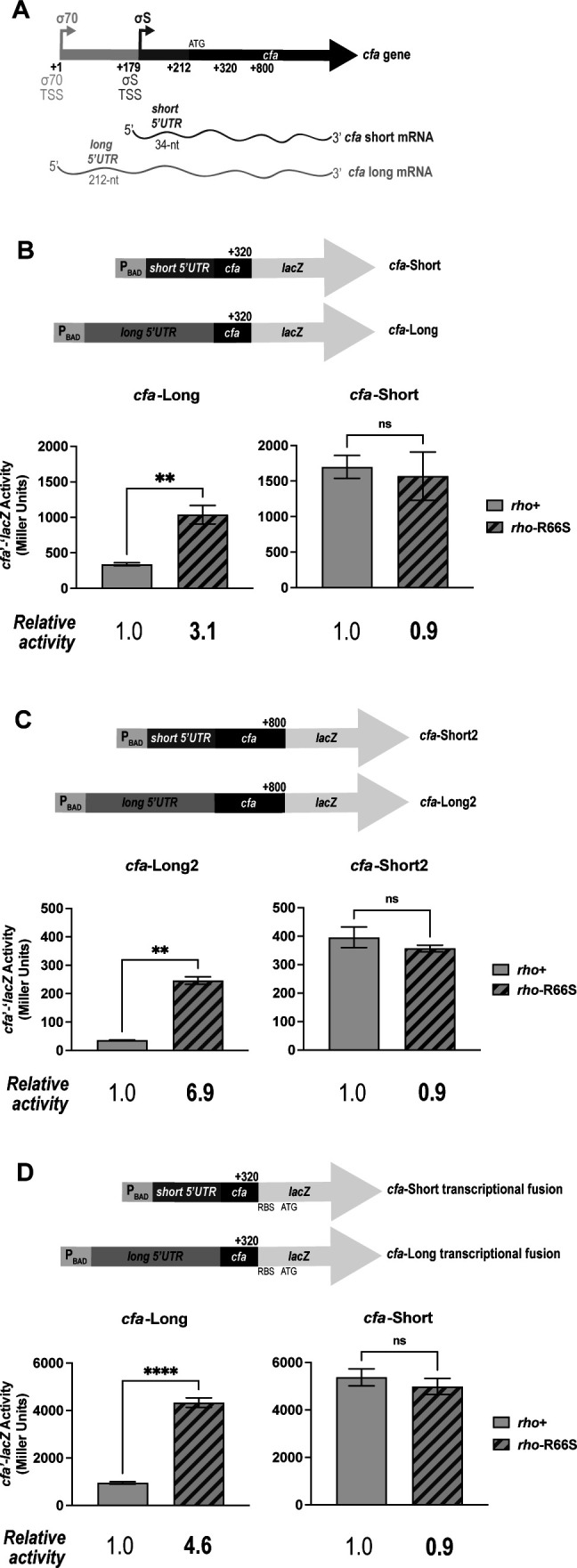
The long 5′ UTR of *cfa* mRNA is required for Rho-dependent regulation. (**A**) The long *cfa* mRNA isoform contains a 212-nt 5′ UTR (*cfa*-Long), while the short *cfa* mRNA isoform contains a 34-nucleotide 5′ UTR (*cfa*-Short). (**B**) *cfa*-Long and *cfa*-Short translational fusions contain the UTRs and the first 36 codons of the *cfa* coding sequence (to position +320 nt) fused to the ninth codon of the *lacZ* CDS (top). Both fusions are controlled by an arabinose-inducible promoter (P_BAD_). β-galactosidase activity of fusion strains was measured at the mid-exponential phase. Error bars represent the standard deviations of three biological replicates, and statistical significance was determined using two-tailed Welch’s *t*-tests by comparing the fusion activity in the *rho*^+^ strain versus the *rho*-R66S mutant (**P < 0.05). (**C**) *cfa*-Long2 and *cfa*-Short2 fusions contain the UTR regions with the first 196 codons (to +800 nt) of the *cfa* CDS fused to the ninth codon of the *lacZ* CDS (top). β-galactosidase activity was measured, and data were analyzed as described in panel **B**. (**D**) *cfa*-Long and *cfa*-Short transcriptional fusion constructs contain the same fragments as the corresponding translational fusions in panel **B**. β-galactosidase activity was measured, and data were analyzed as described in panel **B**. (****P < 0.0001).

The *cfa* gene is transcribed from two promoters that produce isoforms with either a long (212 nt) or short (34 nt) 5′ UTR (18) (Fig. 1A). To determine if either isoform is subject to premature Rho-dependent termination, we measured the activity of *cfa* reporter fusions in *rho*^+^ and *rho*-R66S mutant strains. (Rho-R66S has a transcription termination defect due to reduced RNA binding [19]) Rho autoregulates its own transcription, and activity of a *rho*'-'*lacZ* fusion was higher in the *rho*-R66S mutant compared to the *rho^+^* strain (Fig. S1), indicating that the *rho-*R66S mutation has the expected effect on Rho activity (20). The activity of a *cfa*-Short translational fusion was similar in both *rho^+^* and *rho*-R66S backgrounds, whereas the activity of the *cfa*-Long fusion was higher in the *rho*-R66S mutant (Fig. 1B).

**Fig 2:**
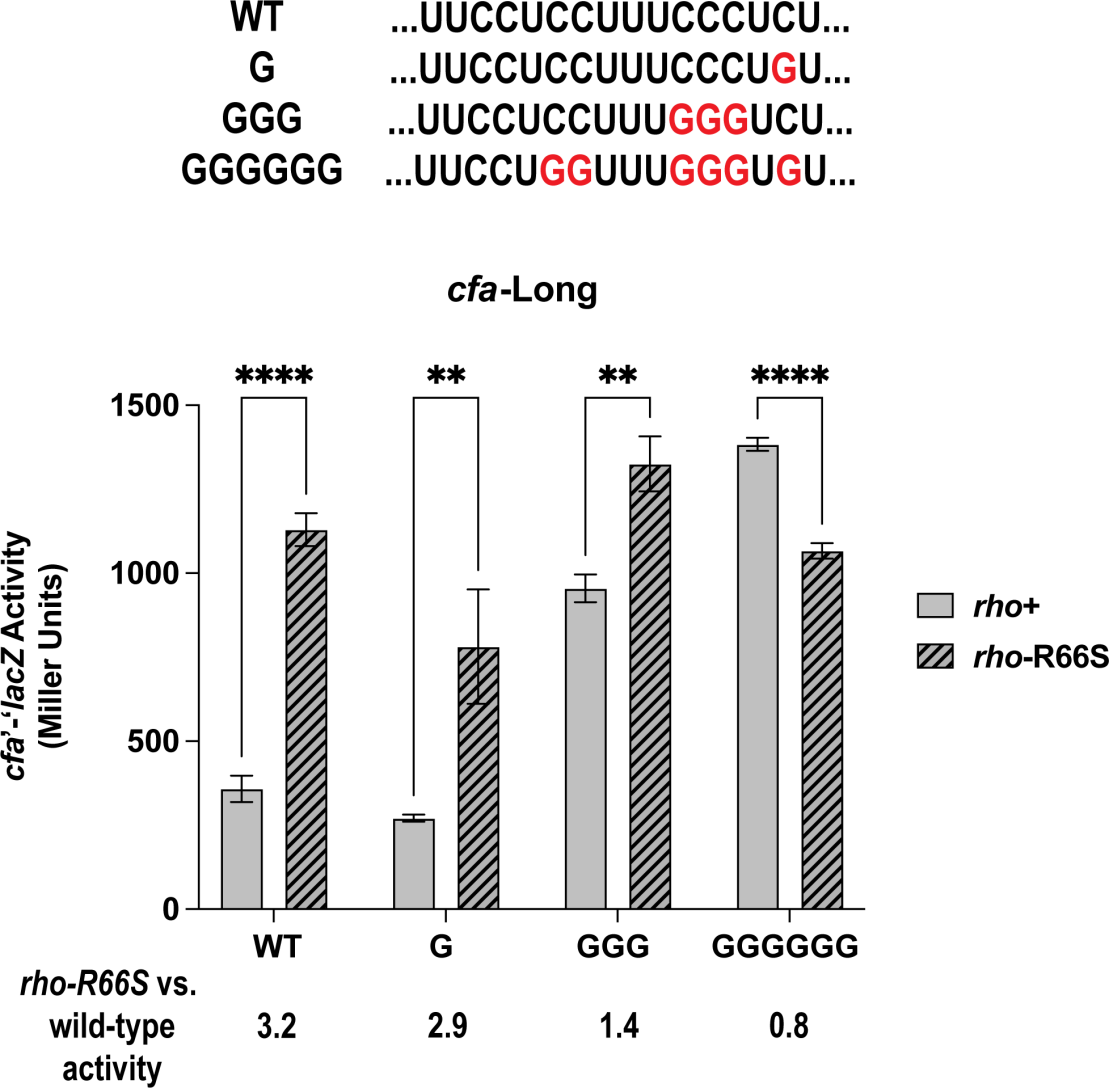
A pyrimidine-rich tract in the *cfa* long 5′ UTR is required for Rho-dependent regulation. The CU-rich tract identified within the *cfa* long 5′ UTR is shown. Variant fusions have C:G substitutions as shown in red font. The β-galactosidase activity of each fusion was tested in *rho ^+^* and *rho-*R66S mutant strain backgrounds. β-galactosidase activity is expressed in Miller Units, and the activity was measured at the mid-exponential phase. Error bars represent the standard deviations of three biological replicates, and statistical significance was determined using multiple unpaired Welch’s *t*-tests by comparing the activity in the wild-type strain versus the *rho*-R66S mutant (****P < 0.0001).

Multiple *cfa* mRNA 3′ ends have been identified (17). One is at position +59 (relative to the long isoform start site), another is at position +330 within the *cfa* coding sequence, and a third bicyclomycin-dependent 3′ end is at +446, suggesting a putative Rho-dependent termination event near this site (17). We hypothesized that Rho binds to a *rut* site in the *cfa* long 5′ UTR and terminates transcription within the 5′ UTR or CDS. To determine if sequences in the *cfa* CDS influence regulation, we constructed *cfa*'*-*'*lacZ* fusions that extend to position +800 (*cfa*-Long2, *cfa*-Short2, Fig. 1C). Activity of the *cfa*-Short2 fusion was similar in *rho^+^* and *rho*-R66S mutant strains, while the activity of the *cfa*-Long2 fusion was significantly higher in the *rho*-R66S mutant (Fig. 2C). The fold change in activity in *rho^+^* compared to *rho-*R66S is higher for the *cfa-*Long2 than for the *cfa*-Long fusion (compare Fig. 1B and C), suggesting the presence of additional sites of Rho-dependent termination in the *cfa-*Long2 compared with *cfa-*Long fusion. Activity of analogous transcriptional fusions showed the same result. There was no Rho-dependent regulation of a *cfa*-Short transcriptional fusion and strong regulation of a *cfa*-Long transcriptional fusion (Fig. 1D).

### A pyrimidine-rich tract in the *cfa* 5′ UTR is required for Rho-dependent regulation

While *rut* sites have no consensus sequence, they typically comprise ~80-nt C-rich/G-poor unstructured regions (6). Some *rut* sites have pyrimidine-rich tracts that promote Rho-dependent termination (21), and we identified a 16-nt CU-rich region between +86 and +101 of the *cfa* long 5′ UTR. To test the role of this region in the regulation of *cfa*, we measured the activity of *cfa*-Long fusion variants with C to G substitutions. While a single substitution (G, Fig. 2) had a minimal impact on activity, three substitutions (GGG, Fig. 2) resulted in strongly increased activity in the *rho^+^* strain, suggesting the GGG variant is less susceptible to Rho-dependent termination. Six substitutions (GGGGGG, Fig. 2) resulted in a further increase in fusion activity in the *rho*^+^ strain, and activity of this fusion was not increased in the *rho*-R66S compared to the *rho^+^* background. This result suggests that Rho cannot promote termination of the GGGGGG variant transcript. These data are consistent with the hypothesis that the 16-nt CU-rich region is part of a larger *rut* site required for Rho-dependent regulation of *cfa*.

Alignment of *cfa* 5′ UTR sequences from 10 different bacterial species belonging to the Enterobacteriaceae (Fig. S2A) revealed strong conservation of the CU-rich region (Fig. S2A). Since *cfa* in *Salmonella enterica* is regulated by some of the same factors as *E. coli cfa* (12, 16), we tested whether *S. enterica cfa* is regulated by Rho. The activity of *S. enterica cfa*-Long and *cfa*-Short translational fusions (*Stm* fusions, Fig. S2B) in the *E. coli* wild-type and *rho*-R66S mutant backgrounds showed that *Stm cfa*-Long fusion activity is higher in the *rho*-R66S mutant compared to the wild-type strain (Fig. S2B). These data suggest that Rho-dependent regulation of *cfa* is conserved between *E. coli* and *Salmonella*.

**Fig 3:**
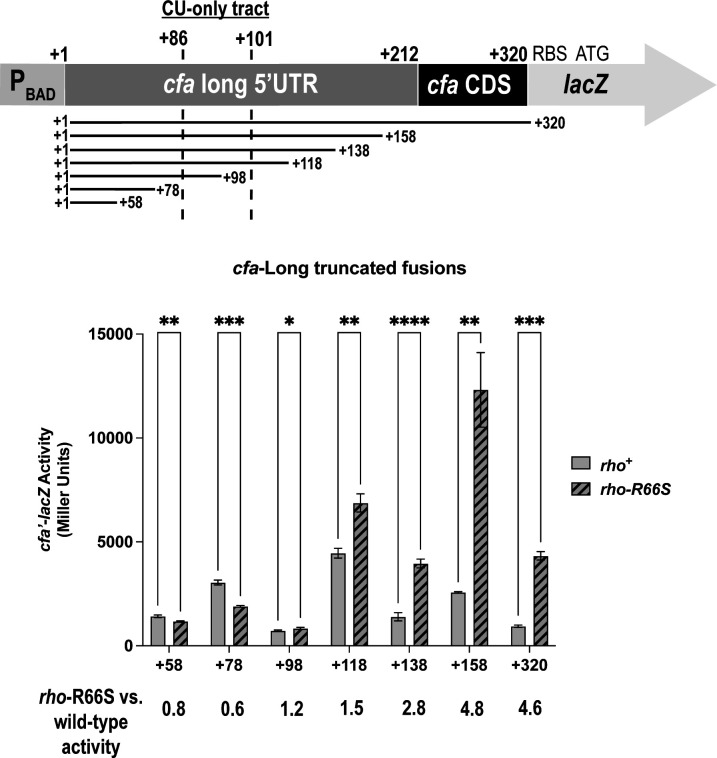
The *cfa* long 5′ UTR contains all sequences needed for Rho-dependent regulation. Six *cfa*-Long transcriptional fusions that were truncated from the 3′ end were constructed (top). The β-galactosidase activity of each truncated fusion was tested in the *rho^+^* and *rho-*R66S mutant strain background (bottom). β-galactosidase activity is expressed in Miller Units, and the activity was measured at the mid-exponential phase. Error bars represent the standard deviations of three biological replicates, and statistical significance was determined using multiple unpaired Welch’s *t*-tests by comparing the activity in the wild-type strain versus the *rho*-R66S mutant (****P < 0.0001).

**Fig 4:**
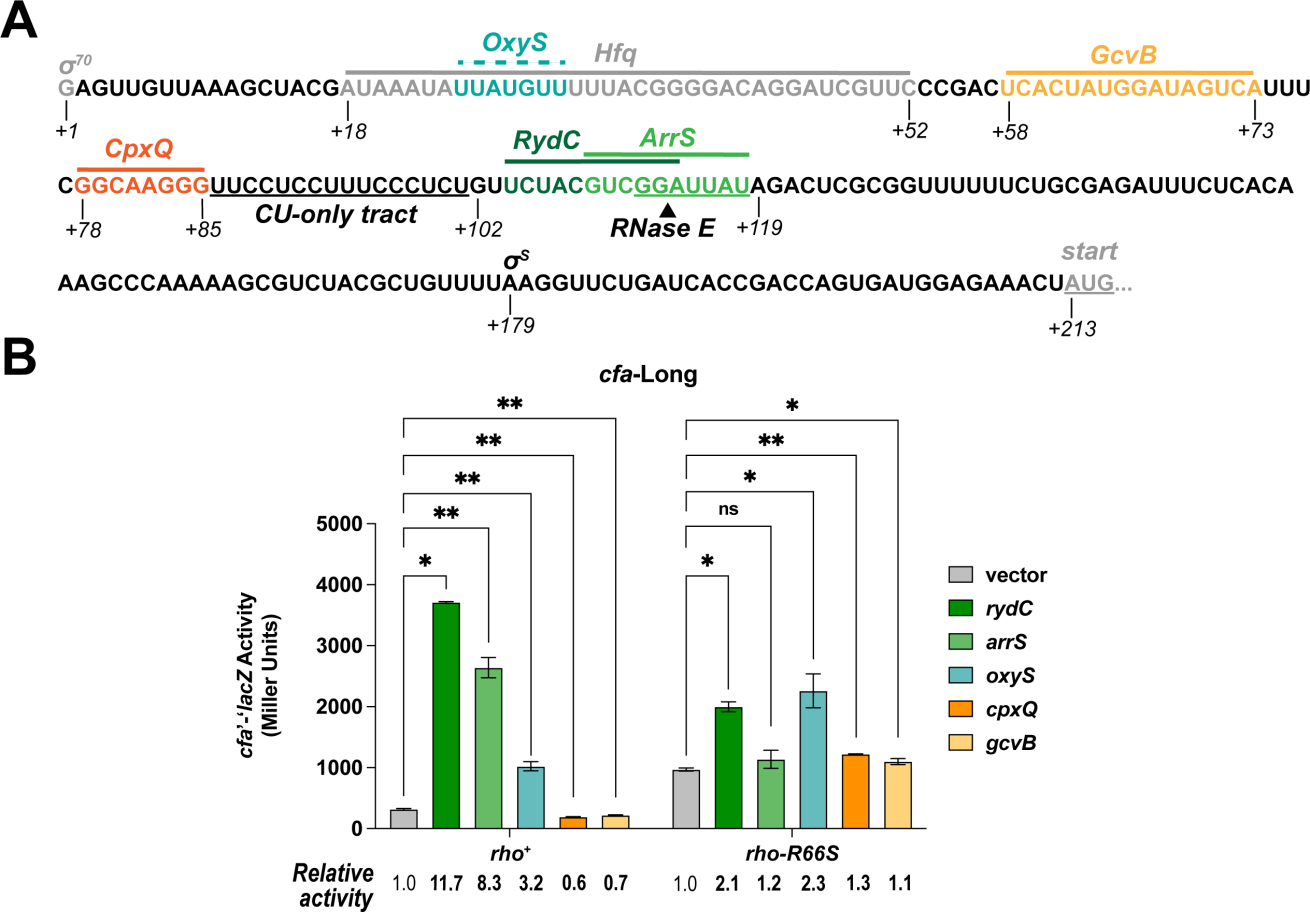
Activating and repressing sRNAs modulate Rho-dependent regulation of *cfa* mRNA. (**A**) The *cfa* σ^70^ transcription start site position is +1, and binding sites are indicated with solid lines (for validated binding sites) or dotted lines (for predicted sites) above the sequence. Validated and predicted binding sites for Hfq and sRNAs were initially reported in reference 11. The predicted RNase E cleavage site and CU-rich region are underlined. The arrow indicates the predicted RNase E cleavage point. (**B**) β-galactosidase activity of the *cfa*-Long translational fusion in the *rho*^+^ or *rho-*R66S mutant strain background in the presence of vector control or plasmids producing the sRNAs RydC, ArrS, OxyS, CpxQ, or GcvB. β-galactosidase activity is expressed in Miller Units, and the activity was measured at the mid-exponential phase after 1 hour of sRNA induction. Error bars represent the standard deviations of three biological replicates, and statistical significance was determined using multiple unpaired Welch’s *t*-tests (**P < 0.05).

To further characterize determinants of Rho-dependent termination, we measured the activity of truncated *cfa*-Long transcriptional fusions (Fig. 3). While basal activities of the fusions varied, fusions containing the 5′ UTR with sequences at or beyond +138 displayed more than twofold higher levels of activity in the *rho*-R66S compared to the *rho^+^* strain (Fig. 3). Shorter fusions (+58, +78, +98, and +118) showed little or no Rho dependence (Fig. 3). The CU-rich region is located between positions + 85 and +102 (Fig. 4A). These data indicate that sequences downstream of this region are also required for efficient Rho-dependent termination in the *cfa* 5′ UTR. Thus, the CU-rich region and sequences immediately downstream constitute a *rut* site required for Rho-dependent termination of *cfa* transcription.

### Activating and repressing sRNAs modulate Rho-dependent *cfa* regulation

The long *cfa* mRNA isoform is regulated by several Hfq-dependent sRNAs. RydC, ArrS, and OxyS activate *cfa*, while CpxQ and GcvB repress *cfa* (11). RydC and ArrS bind to overlapping sites (Fig. 4A), and previous work suggested that they occlude an RNase E cleavage site and impair degradation of *cfa* mRNA (11, 12, 16). CpxQ binds upstream of the RydC binding site (Fig. 4A), and we reported that CpxQ regulates *cfa* by a mechanism requiring the same RNase E cleavage site (11). RydC and ArrS bind immediately downstream of the CU-rich region (within the window required for efficient Rho-dependent termination), while CpxQ binds directly upstream of this region (Fig. 4A). We hypothesized that some or all of these sRNAs modulate Rho-dependent termination of *cfa* transcription.

We measured the activity of the *cfa*-Long fusion in *rho*^+^ and *rho*-R66S mutant strains carrying a vector control or sRNA expression plasmid (Fig. 4B). These sRNAs do not regulate the *cfa*-Short fusion (11). RydC and ArrS activate *cfa*-Long fusion activity in the *rho*^+^ strain, while CpxQ represses it. In the *rho*-R66S mutant, the magnitude of RydC-dependent activation of *cfa* is strongly diminished, and ArrS-dependent activation and CpxQ-dependent repression are completely abolished in this background (Fig. 4B). Northern blot analysis showed that RydC and CpxQ levels are equivalent in *rho*^+^ and *rho*-R66S mutant strains (Fig. S3). We observed the same patterns of sRNA- and Rho-dependent regulation for the *cfa*-Long2 translational fusion and *cfa*-Long transcriptional fusion (Fig. S4). GcvB binds upstream of the CpxQ binding site (11) and modestly represses *cfa-*Long (Fig. 4A); repression is abolished in the *rho*-R66S mutant strain (Fig. 4B). These data suggest that RydC, ArrS, GcvB, and CpxQ regulate *cfa* by a mechanism involving Rho-dependent termination. OxyS modestly activates the *cfa-*Long fusion (11), and the predicted OxyS binding site is far upstream of the CU-rich region (Fig. 4A). OxyS-mediated activation is only slightly diminished in the *rho*-R66S mutant compared to the *rho*^+^ strain (Fig. 4B), suggesting that OxyS activation of *cfa* does not depend solely on Rho-dependent termination. For further experiments to determine the mechanism of sRNA-mediated control of Rho-dependent termination, we focused on one positive (RydC) and one negative (CpxQ) regulator of *cfa*.

### RydC and CpxQ impact *cfa* transcription elongation

**Fig 5:**
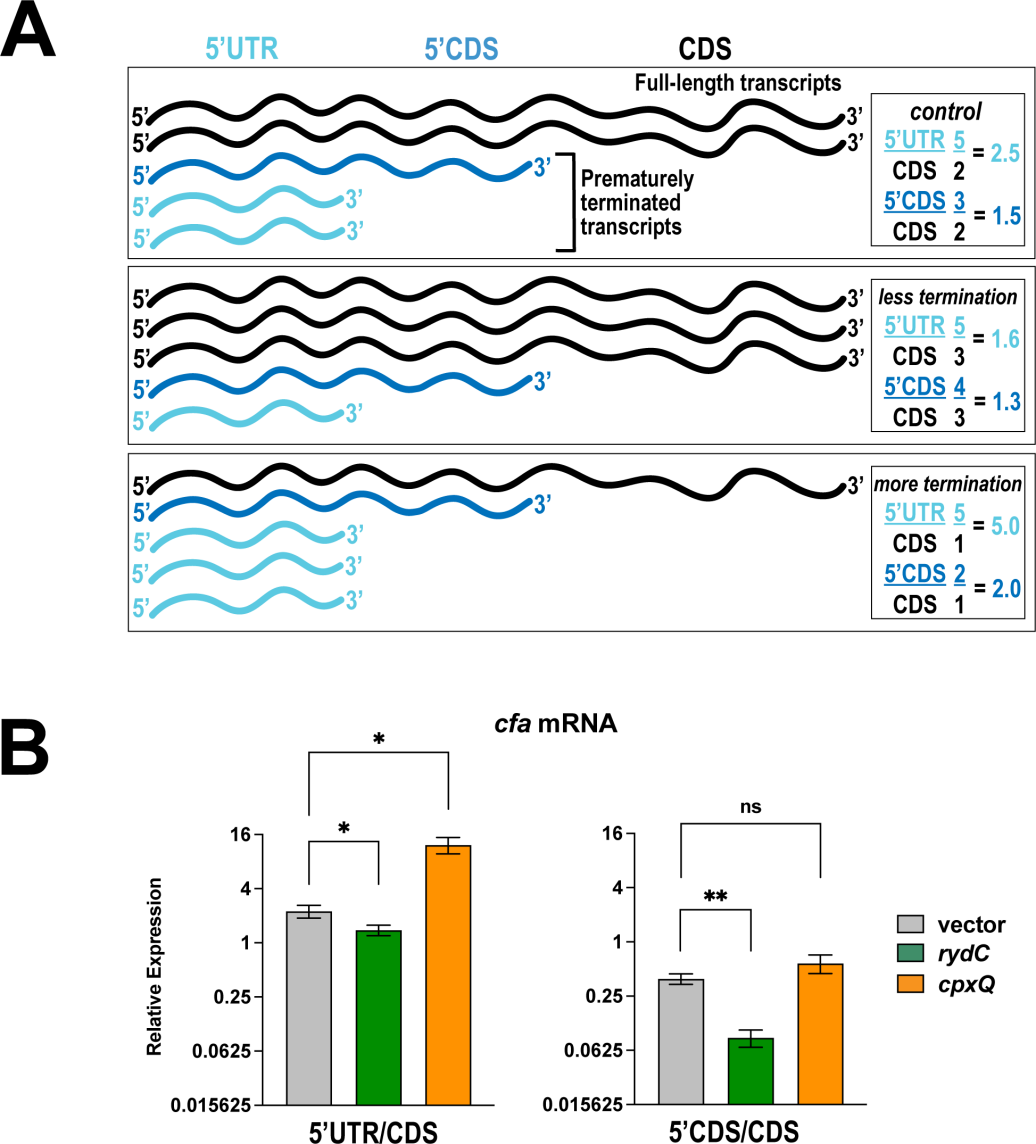
RydC and CpxQ modulate the production of full-length *cfa* mRNA. (**A**) Predictions for ratios of different regions of a transcript subject to Rho-dependent termination with increased or decreased efficiency of Rho-dependent termination. (**B**) RT-qPCR analysis was used to measure the relative levels of different regions of the *cfa* mRNA in Δ*cpxQ* Δ*rydC* mutant cells containing an empty vector or plasmid expressing either RydC or CpxQ. The relative levels of three different regions of *cfa* mRNA were measured, including the long 5′ UTR (5′ UTR), the 5′ portion of the CDS (5′ CDS), and the 3′ portion of the CDS (CDS). Error bars represent the standard deviations of three biological replicates, and statistical significance was determined using multiple unpaired Welch’s *t*-tests (**P < 0.05).

To examine the roles of RydC and CpxQ in modulating premature termination, we used reverse transcription and quantitative PCR (RT-qPCR) to measure the relative levels of different portions of *cfa* mRNA in Δ*rydC* Δ*cpxQ* mutant cells expressing either RydC or CpxQ. Probes were specific for the *cfa* 5′ UTR, the 5′ CDS, and the 3′ CDS (CDS). Figure 5A illustrates how ratios of the PCR products change with increased or decreased termination. When RydC was produced, the *cfa* 5′ UTR:CDS ratio was slightly decreased (Fig. 5B, left panel), and the *cfa* 5′ CDS:CDS ratio was significantly decreased relative to the control (Fig. 5B, right panel), consistent with RydC inhibition of premature termination. The data suggest that RydC promotes elongation past the 5′ region of the CDS and into the 3′ region of the *cfa* CDS. In CpxQ-producing cells, the *cfa* 5′ UTR:CDS ratio is increased, consistent with a CpxQ-mediated increase in termination (Fig. 5B). CpxQ-producing cells did not display a significant change in 5′ CDS:CDS ratios compared to the control. These data suggest that CpxQ modulates termination in the region between the 5′ UTR and 5′ CDS probes. These data support the model that CpxQ and RydC modulate *cfa* transcription elongation.

### CpxQ binding to a stem-loop in the *cfa* 5′ UTR governs *rut* site accessibility

**Fig 6:**
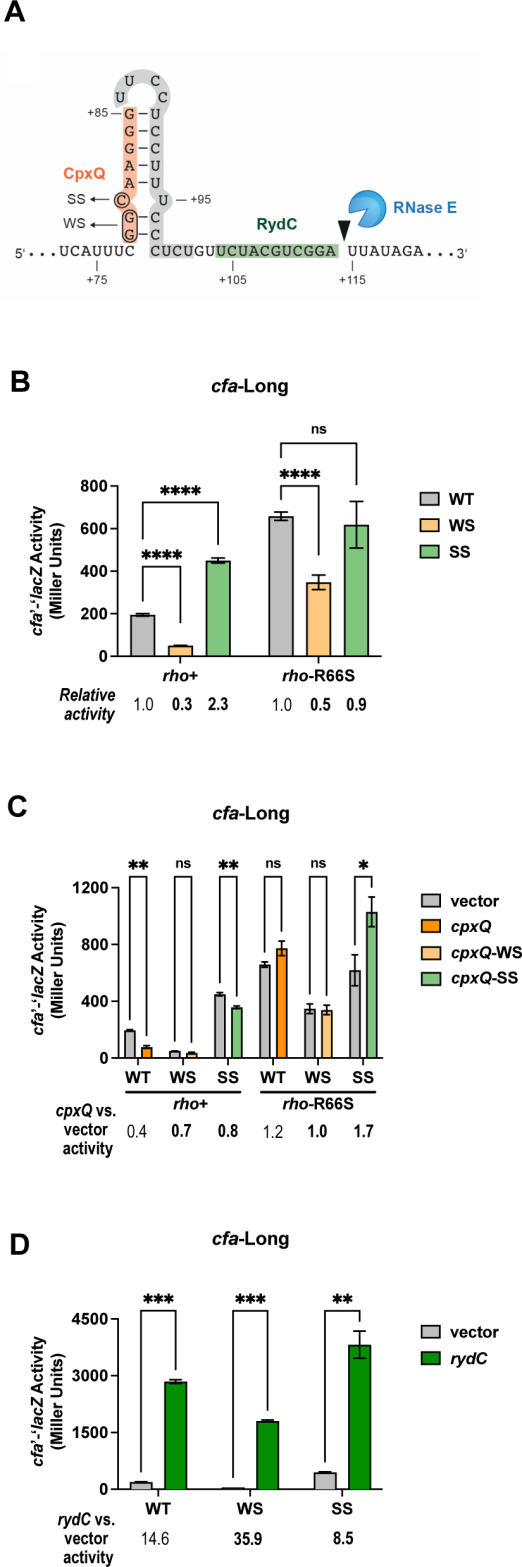
CpxQ enhances *rut* site accessibility by preventing stem-loop formation within the *cfa* long 5′ UTR. (**A**) The stem-loop structure in the *cfa* long 5′ UTR that encompasses the CU-rich portion of the putative *rut* site (shaded gray) and the CpxQ binding site (shaded orange). The RydC binding site (shaded green) and a putative RNase E cleavage site are indicated. (**B**) *cfa*-Long translational fusion variants: weak stem (WS) disrupts the stem-loop via two G:C substitutions, and strong stem (SS) reinforces the stem-loop via a C:A substitution. Activity of wild-type (WT) *cfa*-Long, WS, and SS variant fusions was tested in *rho^+^* and *rho-*R66S mutant strain backgrounds. β-galactosidase activity is expressed in Miller Units, and the activity was measured at the mid-exponential phase. Error bars represent standard deviations of three biological replicates; statistical significance was determined using multiple unpaired Welch’s *t*-tests (****P < 0.0001). (**C**) Activity of WT, WS, and SS *cfa*-Long fusions in *rho^+^* and *rho-*R66S mutant strain backgrounds carrying vector control or plasmids producing CpxQ, CpxQ-WS, or CpxQ-SS. β-galactosidase activity was measured as described in panel **B** after 1 hour of sRNA induction. Data were analyzed as described in panel **B** (**P < 0.05). (**D**) β-galactosidase activity of WT, WS, and SS *cfa*-Long fusions in *rho^+^* and *rho-*R66S mutant strain backgrounds carrying vector control or RydC-producing plasmids. β-galactosidase activity was measured as described in panel **B** after 1 hour of sRNA induction. Data were analyzed as described in panel **B** (***P < 0.001).

Other sRNAs that modulate Rho-dependent termination within 5′ UTRs are thought to inhibit Rho binding to the mRNA, leading to increased transcription elongation (7, 10). In contrast, CpxQ increases Rho-dependent termination in the *cfa* 5′ UTR (Fig. 5B). We hypothesized that CpxQ binding to *cfa* mRNA alters its secondary structure to increase the accessibility of the *rut* site. We found a putative stem-loop structure from +78 to +97 of the 5′ UTR (Fig. 6A). The 5′ half of the stem contains the CpxQ binding site, while the loop and 3′ half of the stem encompass the pyrimidine-rich region that is critical for Rho-dependent termination (Fig. 6A). We predicted that formation of this stem-loop occludes the *rut* site and limits Rho-dependent termination. Binding by CpxQ would prevent formation of the stem, leaving the *rut* site more accessible and leading to increased Rho-dependent termination.

To test the role of this stem in the regulation of *cfa*, we designed *cfa*-Long translational fusions to either weaken or strengthen the stability of the stem (Fig. 6A). The *cfa*-Long-G78C-G79C fusion, referred to as *cfa*-WeakStem (*cfa-*WS), should disrupt stem formation and increase Rho-dependent termination. The *cfa*-Long-C80A fusion, referred to as *cfa*-StrongStem (*cfa*-SS), should strengthen the stem, leading to decreased Rho-dependent termination. We measured the activity of *cfa*-Long, *cfa*-WS, and *cfa*-SS and found that *cfa*-WS exhibited a significant decrease in activity compared with *cfa*-Long (Fig. 6B), consistent with increased Rho access to the *rut* site and increased termination. In contrast, the *cfa*-SS fusion displayed an approximately twofold increase in activity compared with *cfa*-Long (Fig. 6B), consistent with reduced Rho access to the *rut* site and reduced termination. Consistent with the hypothesis that differences in reporter activity are due to differences in Rho-dependent termination, the impact of the stem-weakening and stem-strengthening mutations was either attenuated or abolished in the *rho*-R66S mutant background (Fig. 6B).

We hypothesized that the WS and SS mutations would blunt the impact of CpxQ on *cfa*. CpxQ variants with compensatory mutations that restore base pairing with *cfa-*WS (CpxQ-WS) and *cfa-*SS (CpxQ-SS) were expressed in *rho*^+^ and *rho-*R66S backgrounds. In contrast with the wild-type interaction, which inhibits *cfa* fusion activity, there is no statistically significant repression by CpxQ-WS or CpxQ-SS on the relevant fusions in the *rho*^+^ background. In the *rho-*R66S background, there was no CpxQ-mediated repression, though we did observe a small CpxQ-SS-mediated activation of *cfa*-SS that we cannot explain (Fig. 6C).

The RydC binding site is downstream of the CU-rich stem-loop, within the region (+1 to +138) important for Rho-dependent regulation of *cfa* (Fig. 3). We hypothesize that RydC directly occludes Rho binding by blocking part of the *rut* site downstream of the CU-rich stem-loop. If this is true, we expected that RydC-dependent activation of *cfa* is independent of the formation of the stem-loop. To test this hypothesis, we measured the activity of *cfa-*Long, *cfa-*WS, and *cfa-*SS fusions in strains carrying a vector control or *rydC* expression plasmid. As predicted, RydC strongly activated all three fusions (Fig. 6D). Together, these data are consistent with the model that repressing and activating sRNAs regulate *cfa* by modulating access of Rho to different portions of a *rut* site in the *cfa* 5′ UTR.

### Interplay between Rho and RNase E degradosome in the regulation of *cfa*

**Fig 7:**
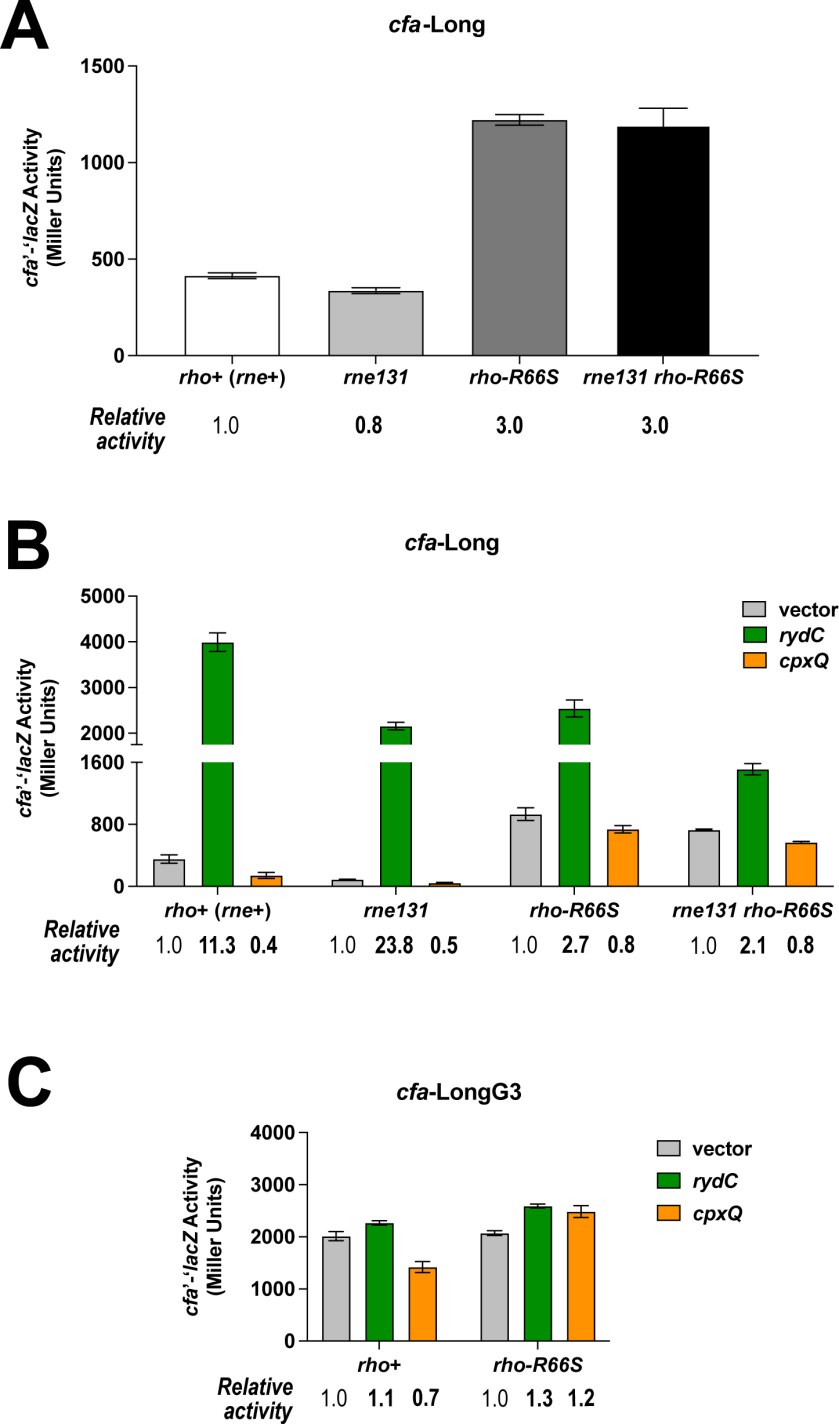
Activating and repressing sRNAs primarily regulate Rho-dependent regulation of *cfa* mRNA. (**A**) Activity of the *cfa*-Long translational fusion in wild-type (*rho*^+^*rne*^+^), *rne131* mutant, *rho*-R66S mutant, and *rne131 rho*-R66S mutant strains. β-galactosidase activity was measured at the mid-exponential phase and is expressed in Miller Units. Error bars represent the standard deviations of three biological replicates. (**B**) Activity of the *cfa* Long translational fusion in the same strains as in panel **A**, carrying vector control or plasmids producing RydC or CpxQ. β-galactosidase activity is expressed in Miller Units, and the activity was measured at the mid-exponential phase after 1 hour of sRNA induction. Error bars represent the standard deviations of three biological replicates. (**C**) Activity of the *cfa*-LongG3 translational fusion in the *rho*^+^ or *rho*-R66S mutant strain background carrying a vector control or plasmid expressing RydC or CpxQ. Error bars represent the standard deviations of three biological replicates.

Previous studies in *S. enterica* and *E. coli* showed that *cfa* mRNA turnover is regulated by RNase E cleavage within the 5′ UTR (12, 16) and suggested that RydC and CpxQ inhibit or enhance cleavage, respectively (11). We measured *cfa*-Long fusion activity in *rne131* strains that produce a degradosome-deficient RNase E variant (22), which cannot carry out many sRNA-dependent regulatory effects (23–25). The activity of the fusion was similar in *rne^+^* and *rne131* mutant backgrounds and was approximately threefold higher in the *rho-*R66S mutant. Activity in the *rho-*R66S *rne131* double mutant was similar to the *rho-*R66S mutant (Fig. 7A), suggesting that degradosome-mediated decay does not contribute significantly to *cfa* regulation. RydC and CpxQ regulate the *cfa*-Long fusion similarly in wild-type and *rne131* single mutant strains (Fig. 7B). In the *rho-*R66S background, CpxQ-dependent repression and RydC-dependent activation are both diminished compared to *rho*+ strains. Regulation by sRNAs is similar between *rho*-R66S single and *rho*-R66S *rne131* double mutant strains (Fig. 7B). These results suggest that RydC and CpxQ primarily regulate *cfa* by modulating Rho-dependent termination and not degradosome activity.

Previous work demonstrated that *Salmonella cfa* mRNA is cleaved by RNase E at a position adjacent to the RydC binding site (16). Mutation of the cleavage site increased *cfa* mRNA levels and made *cfa* less sensitive to RydC-mediated regulation (12). We made a similar mutation in the *E. coli cfa* 5′ UTR (GG↓AUUAU to GG↓AGGGU, where the arrow indicates the site of cleavage) and showed that the mutation increased *cfa* fusion activity and diminished regulation by RydC and CpxQ (11). Our original interpretation was that both RydC and CpxQ modulate RNase E activity on *cfa* mRNA. However, in light of our current results, we hypothesized that the mutation (where UUA is replaced by GGG, which we will call G3 here) impairs Rho-dependent regulation of *cfa*. To test this, we compared the activity of the *cfa*-LongG3 fusion in the *rho^+^* and *rho*-R66S backgrounds carrying an empty vector, RydC-, or CpxQ-producing plasmids (Fig. 7C). The G3 mutation strongly increases basal levels of *cfa* expression (compare activity of *cfa-*Long in Fig. 7A to *cfa-*LongG3 in Fig. 7C) and RydC- and CpxQ-mediated regulation is lost or diminished, respectively (Fig. 7C). In the *rho-*R66S background, there is no further increase in the activity of the *cfa*-LongG3 fusion (Fig. 7C). These data indeed suggest that the G3 mutation disrupts Rho-dependent regulation of *cfa*, and that this explains changes in G3 fusion activity levels and the loss of RydC- and CpxQ-mediated regulation.

### RydC regulates *cfa* even when RNase E is inactivated

**Fig 8:**
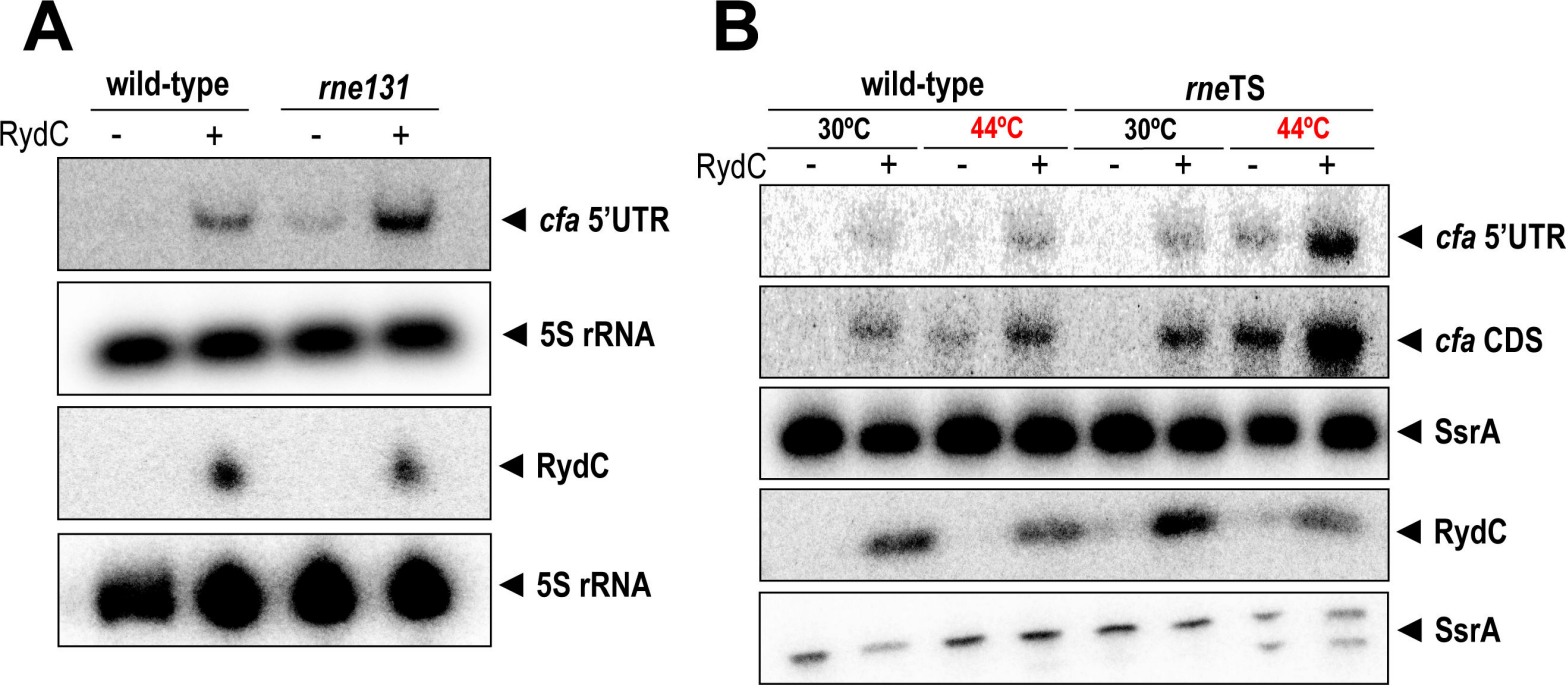
RydC regulates *cfa* even when RNase E is inactivated. (**A**) Northern blot analysis was used to examine *cfa* mRNA levels in wild-type (*rne*^+^) and RNase E degradosome mutant (*rne131*) cells in the presence or absence of RydC. The 5S rRNA was used as an RNA loading control. (**B**) Northern blot analysis was used to examine *cfa* mRNA levels in wild-type (*rne*^+^) and RNase E temperature-sensitive mutant (*rne*TS) cells at permissive (30°C) and non-permissive (44°C) temperatures in the presence or absence of RydC.

To further probe the role of RNase E in the regulation of *cfa* in *E. coli*, we measured *cfa* mRNA levels in *rne*^+^ and *rne131* mutant strains carrying vector control or RydC expression plasmids. In the *rne*^+^ strain with the vector control, *cfa* mRNA was undetectable, and levels increased substantially in the strain producing RydC (Fig. 8A). In the *rne131* degradosome mutant, *cfa* mRNA levels were low but detectable, and RydC substantially increased these levels (Fig. 8A). These results suggest that the degradosome plays a minor role in *cfa* mRNA turnover, and RydC increases *cfa* mRNA levels by a degradosome-independent mechanism.

We next compared *cfa* mRNA levels in *rne^+^* and a strain with a temperature-sensitive RNase E (*rne*Ts) (26) each carrying a vector control or RydC expression plasmid (Fig. 8B). In the *rne*^+^ strain grown at both 30°C and 44°C, *cfa* mRNA levels increased when RydC was produced (Fig. 8B). In the *rne*Ts strain grown at 30°C, *cfa* mRNA was only detected when RydC was produced. At 44°C, *cfa* mRNA was detectable in the absence of RydC, and levels were further increased when RydC was produced (Fig. 8B). If RydC were activating *cfa* primarily by antagonizing RNase E activity, there would be no RydC-dependent accumulation of *cfa* mRNA at the non-permissive temperature where RNase E is inactive. Our data suggest that while *cfa* mRNA is subject to RNase E-mediated turnover, RydC regulates *cfa* primarily by modulating Rho-dependent transcription termination.

## DISCUSSION

**Fig 9:**
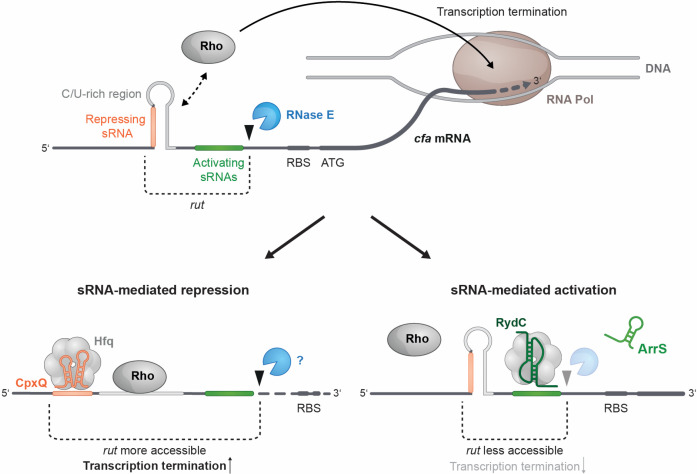
Model for *cfa* regulation by Rho and sRNAs. The model is described in detail in Discussion. Both activating and repressing sRNAs modulate Rho-dependent transcription termination of *cfa* mRNA. A stem-loop structure containing both the CpxQ binding site and the CU-rich region, which is required for Rho-dependent termination and is most likely part of a larger *rut* site, was identified in the long *cfa* 5′ UTR. The RydC and ArrS binding sites are immediately downstream of the stem-loop structure. CpxQ binding diminishes stem-loop formation, leading to increased *rut* site accessibility and Rho-dependent termination to repress *cfa* transcription. RydC (or ArrS) binding enhances stem-loop formation, leading to decreased *rut* site accessibility and Rho-dependent termination to activate *cfa* transcription. RydC may also impair RNase E-dependent decay of *cfa* mRNA.

The cyclopropane fatty acid (*cfa*) synthase mRNA is one of a handful of mRNA targets that are hubs for the integration of environmental signals via multiple sRNA regulators. Regulation of *cfa* by multiple sRNAs (11, 12, 16) is particularly intriguing from a mechanistic standpoint because the sRNAs bind at sites distant from the translation initiation region and mediate both positive and negative regulation by translation-independent mechanisms. In this study, we uncovered new details of these mechanisms that expand our understanding of the varied ways that sRNAs regulate bacterial gene expression. Our data are consistent with a model (Fig. 9) where transcription of the long *cfa* isoform is limited by Rho. The long 5′ UTR of *cfa* mRNA contains a *rut* site that includes a CU-rich region required for Rho-dependent regulation. Accessibility of the *rut* site is altered by the presence of a stem-loop that partially sequesters the CU-rich portion of the *rut* site. CpxQ can repress *cfa* by preventing stem-loop formation, making the *rut* site more accessible and increasing transcription termination (Fig. 9, bottom left). RydC binds to a site downstream of the stem-loop, sequestering a different part of the *rut* site, leading to decreased transcription termination (Fig. 9, bottom right). The sRNAs may also play a minor role in modulating RNase E-mediated *cfa* mRNA turnover, but their major function appears to be control of transcription elongation.

Regulation of bacterial transcription is often thought of as occurring mainly at the level of initiation through transcription factors that affect RNA polymerase binding to promoter sequences. However, both protein and RNA factors can regulate transcription elongation through attenuation (increased termination) or antitermination (decreased termination) mechanisms (27). In *E. coli*, 20%–30% of transcription termination events are Rho-dependent (28, 29). Because Rho requires a single-stranded *rut* site unoccupied by ribosomes, processes that impact translation or RNA structure can influence the efficiency of Rho-dependent termination. Several proteins, including Hfq, have been reported to modulate Rho activity and Rho-dependent termination (30–34). While Hfq is best known as the chaperone involved in sRNA stability and sRNA-mediated regulation, it can also bind directly to Rho to inhibit Rho-dependent termination (32). Small RNAs commonly impact translation and structure of mRNA targets, so it follows that sRNAs can control Rho-dependent termination (35). The first translation-dependent mechanism of sRNA control of Rho termination was reported by Bossi and colleagues in 2012 (21). ChiX sRNA represses translation of *chiP* by sequestering the RBS, which in turn increases the rate of *chiPQ* mRNA turnover (36) and makes a *rut* site in the *chiP* coding sequence more accessible, promoting premature Rho-dependent termination (21). Similarly, the sRNA Spot 42 binds to the *galK* RBS within the polycistronic *galETKM* mRNA. Repressed *galK* translation leads to increased Rho-dependent termination within *galK* (37). Our work demonstrated that sRNAs, including SgrS and RyhB, that repress translation of their targets can also promote Rho-dependent termination (38).

Perhaps even more intriguing are a growing number of sRNAs that modulate Rho-dependent termination by translation-independent mechanisms. Long 5′ UTRs are often sites of sRNA action, and these sequences are unprotected by ribosomes. Hundreds of long 5′ UTRs in *E. coli* may be targets for Rho-dependent termination (7). Multiple sRNAs activate *rpoS* translation by preventing the formation of a translation-inhibitory hairpin in the 5′ UTR (39–42). These sRNAs were also shown to antagonize premature Rho-dependent termination within the 5′ UTR (7). In *Salmonella*, SraL base pairs with the 5′ UTR of *rho* mRNA to protect it from auto-regulated termination (10). For both *rpoS* and *rho*, the mechanism of sRNA-mediated interference with Rho-dependent termination within 5′ UTRs is unclear. To the best of our knowledge, this work is the first to report an sRNA that mediates increased Rho-dependent termination of its mRNA target. Our results demonstrate that CpxQ binding within the 5′ portion of a stem-loop partially sequesters sequences that are part of a *rut* site and increases Rho-dependent termination. This is reminiscent of a mechanism used by the RNA-binding protein CsrA, which promotes Rho-dependent termination by binding to the *pgaA* mRNA 5′ UTR and remodeling the secondary structure of the 5′ UTR to expose a *rut* site (43).

Classical examples of sRNA-mediated regulation involve direct translational repression through base-pairing interactions that occlude the translation initiation region (44). sRNA-mRNA interactions that occur within a window of approximately 40 nt around the start codon can directly inhibit ribosome binding (45). We now know that sRNAs can also carry out translational regulation by base pairing at sites that are long distances from translation initiation regions. We and others have described repression mechanisms involving sRNA sequestration of translation enhancer sequences (46–48) and ribosome standby sites (49) in long 5′ UTRs. Small RNAs can act as indirect translational repressors by binding upstream (50) or downstream (51) of the translation initiation region and recruiting Hfq to bind at sites overlapping the RBS. Perhaps not surprisingly, regulatory mechanisms that act at steps of gene expression other than translation also involve sRNA-mRNA interactions at sites far from the mRNA translation initiation region. Control of mRNA decay can be modulated by sRNAs binding within 5′ or 3′ UTRs or coding regions (52–54). We can now definitively add transcription elongation to the list of molecular processes that sRNAs can modulate by base pairing at sites distal to the translation initiation region. More work will be required to determine how common sRNA-dependent modulation of transcription elongation is and how both sRNAs and Hfq modulate Rho binding to mRNAs and Rho translocation.

## MATERIALS AND METHODS

### Bacterial strains, media, and growth conditions

Bacterial strains and plasmids are listed in Table S1. All strains are derivatives of *E. coli* K-12 MG1655. Bacterial complete growth media were purchased from Research Products International; individual medium components were purchased from Fisher Scientific. Bacteria were cultured in LB broth or on LB agar plates at 37°C unless otherwise specified. Antibiotics and other compounds (from GoldBio unless otherwise specified) were used at the following final concentrations: 100 µg/mL ampicillin, 25 µg/mL chloramphenicol, 50 µg/mL kanamycin (Fisher Scientific), 10 µg/mL tetracycline, L-arabinose (Sigma-Aldrich), 0.1 mM isopropyl-β-D-1-thiogalactopyranoside (IPTG), and 40 µg/mL 5-bromo-4-chloro-3-indolyl β-D-galactopyranoside.

### Molecular methods

Oligonucleotides (IDT) are listed in Table S2. All reagents and enzymes were purchased from New England Biolabs unless otherwise specified. PCR reactions used Phusion or Q5 High-Fidelity DNA Polymerases. Plasmids were designed using Geneious Prime (version 2020.1.2) and the NEBuilder Assembly Tool (version 2.7.1), and plasmids were constructed using the NEBuilder HiFi DNA Assembly Master Mix. Plasmids were verified with restriction enzyme digestion and sequencing.

### Bacterial strain construction

Transcriptional and translational reporter fusions were constructed using λ Red homologous recombination into strain PM1805 and counterselection against *sacB* as previously described (55). gBlock gene fragments were used in the construction of *cfa* transcriptional fusions. PCR amplicons or gBlock gene fragments were used to construct *cfa'-'lacZ* and *rho'-'lacZ* translational fusions. All reporter constructs were verified with PCR and sequencing.

A chloramphenicol resistance gene (*cat*) was inserted in the intergenic region between *flgL* and *rne* via λ Red homologous recombination to link the *rne-*3071 (ts) allele to the *cat* gene (56). The *rho-*R66S, *rne-*131, *rne-*3071, Δ*cpxQ,* and Δ*rydC* mutations were moved from donor strains to recipient strains via P1 *vir* transduction (57). Plasmid pCP20 encoding FLP recombinase was used to remove antibiotic resistance cassettes from Δ*cpxQ*::kan and Δ*rydC*::kan strains as needed (56).

### β-galactosidase assays

Bacterial strains harboring *lacZ* reporter fusions were cultured at 37°C for 16 hours in tryptone broth medium with arabinose, with or without ampicillin. After 16 hours, bacterial strains were subcultured 1:100 in the same media and grown to the early or mid-exponential phase. If sRNA induction was not required, cultures were harvested and assayed at the mid-log phase. If sRNA induction was required, 0.1 mM IPTG was added at the early exponential phase, and cells were cultured at 37°C for 1 hour more before harvesting and assaying at the mid-log phase. β-galactosidase assays were carried out with chloroform-permeabilized cells as previously described (57), and data are expressed in Miller Units.

### RNA extraction

Total RNA was extracted from cells using the hot phenol method as previously described (58) with the following adjustments. One milliliter of culture was mixed with 1 mL of 65°C lysis solution containing SDS, EDTA, sodium acetate, and UltraPure Phenol:Water (3.75:1, vol/vol) (Invitrogen), and the mixture was shaken at 65°C at 1,400 rpm for 15 minutes. Samples were centrifuged at room temperature at 21,300 *× g* for 10 minutes. The aqueous layer was transferred to a 5PRIME Phase Lock Gel Heavy wax tube (Quantabio) and then extracted with one volume of Phenol:Chloroform:Isoamyl alcohol (25:24:1) (Ambion). The aqueous layer was transferred to a tube containing 1.3 mL of absolute ethanol and placed at −80°C. The next day, the sample was centrifuged at 4°C at 14,000 *× g* for 30 minutes. The pellet was allowed to dry before resuspension in 10–40 μL of RNase-free water. RNA samples were stored at −80°C. RNA samples were treated with TURBO DNase (Invitrogen) according to the manufacturer’s instructions. After DNase digestion, samples were purified using phenol-chloroform extraction. To each reaction, 2 volumes of RNase-free water and 0.2 volumes of sodium acetate were added. After the addition of an equivalent volume of acid phenol:chloroform (pH 4.5) (Ambion), each sample was transferred to a Phase Lock Gel Heavy wax tube. RNA was precipitated and then resuspended as described above. RNA concentrations were measured using the Qubit RNA Broad Range (BR) Assay kit.

### Northern blot analysis

DNA probes (IDT) were radiolabeled at the 5′ end with fresh [γ-^32^P] ATP (Perkin Elmer) using the KinaseMax Kit (Invitrogen) according to the manufacturer’s instructions. Radiolabeled probes were purified using ProbeQuant G-50 Micro Columns (Cytiva) and were stored at −80°C until use. Nylon membranes were stripped and re-probed as previously described (11).

For the analysis of *cfa* mRNA levels, 20 µg RNA samples were denatured at 65°C for 15 minutes in loading buffer (containing MOPS buffer, formaldehyde, and formamide), separated on a 0.8% agarose gel for electrophoresis at 80 V for 2 hours in 1× MOPS buffer, and transferred to a BrightStar-Plus Positively Charged Nylon Membrane (Invitrogen) via capillary transfer overnight. Transferred RNAs were UV crosslinked to the membrane, and the membrane was prehybridized with 10 mL of ULTRAhyb Ultrasensitive Hybridization Buffer (Invitrogen) at 42°C for 45 minutes. Blots were allowed to hybridize with radiolabeled probes overnight at 42°C and were then washed once with 2× SSC/0.1% SDS, once with 0.1× SSC/0.1% SDS, and once with 0.1× SSC for 15 minutes each. For signal detection, blots were exposed to a phosphorimager screen, and the results were visualized using a Typhoon FLA 9500 Phosphorimager (GE).

For analysis of sRNA levels, 10 µg RNA samples were prepared and blotted as previously described (11) with the following modifications. Electroblot transfer was run on ice at 250 mA (constant) for 4 hours with 0.5× TBE buffer. After hybridization, blots were washed once with 2× SSC/0.1% SDS, once with 0.1× SSC/0.1% SDS, and once with 0.1× SSC for 15 minutes at 42°C. For signal detection, blots were exposed to a phosphorimager screen, and the results were visualized using a Typhoon FLA 9500 Phosphorimager (GE).

### Reverse transcription and quantitative PCR

RNA extracts were treated with TURBO DNase (Invitrogen) as described above. Samples were verified to be DNA-free using 40-cycle PCR reactions with each PCR primer pair. The RNA concentration was quantified using the Qubit RNA Broad Range (BR) Kit. RT-qPCR reactions (10 µL) contained 250 ng RNA, forward primer, reverse primer, probe, and Luna Probe One-Step RT-qPCR 4× Mix with UDG (NEB) according to the manufacturer’s instructions. Control reactions contained Luna Probe One-Step RT-qPCR 4× Mix with UDG that was first heated at 95°C for 5 minutes to inactivate RT. RT-qPCR reactions were run on a Bio-Rad CFX Connect Real-Time System with Bio-Rad CFX Manager (version 3.1) software in 0.2 mL 96-well PCR plates (Dot Scientific Inc.) sealed with transparent Microseal B Adhesive Sealer (Bio-Rad). Data were analyzed using the comparative Ct (2^-ΔΔCt^) method.

## SUPPLEMENTAL MATERIAL

Supplemental figures and tables mbio.02921-25-s0001.pdf Fig. S1-S4; Tables S1-S2.

ASM does not own the copyrights to Supplemental Material that may be linked to, or accessed through, an article. The authors have granted ASM a non-exclusive, world-wide license to publish the Supplemental Material files. Please contact the corresponding author directly for reuse.
