# Supplementary material for: Small RNAs positively and negatively control transcription elongation through modulation of Rho utilization site accessibility
Source: mBio. 2025 Oct 31;16(12):e02921-25. doi: 10.1128/mbio.02921-25 (PMC12691595; doi:10.1128/mbio.02921-25)
Supplement: Supplemental figures and tables — Fig. S1-S4; Tables S1-S2. [file mbio.02921-25-s0001.pdf]

Supplementary Material

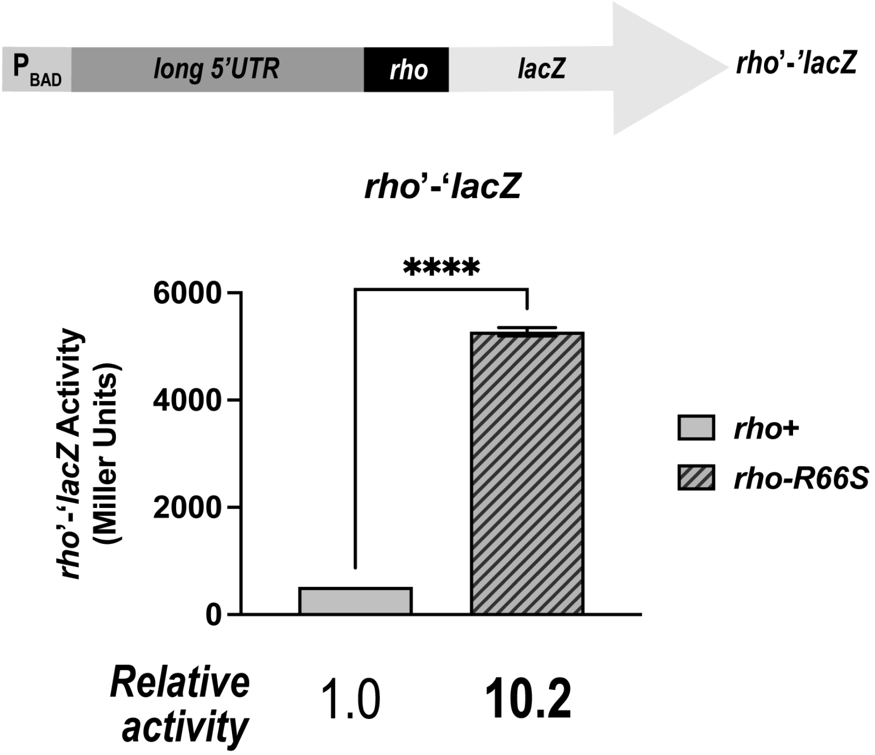

Figure S1. A *rho* reporter fusion responds as expected to the *rho*-R66S allele. A *rho*'-'*lacZ* translational fusion contains the entire 5' UTR of *rho* mRNA and the first 10 codons of the *rho* coding sequence (top). Transcription of the fusion was controlled with an arabinose-inducible promoter (P_{BAD}). β-galactosidase activity of the *rho*'-'*lacZ* fusion was tested in *rho*+ and *rho*-R66S mutant backgrounds (bottom). β-galactosidase activity is expressed in Miller Units and the activity was measured at mid-exponential phase. Error bars represent the standard deviations of three biological replicates and statistical significance was determined using a two-tailed Welch's t-test (****P < 0.0001).

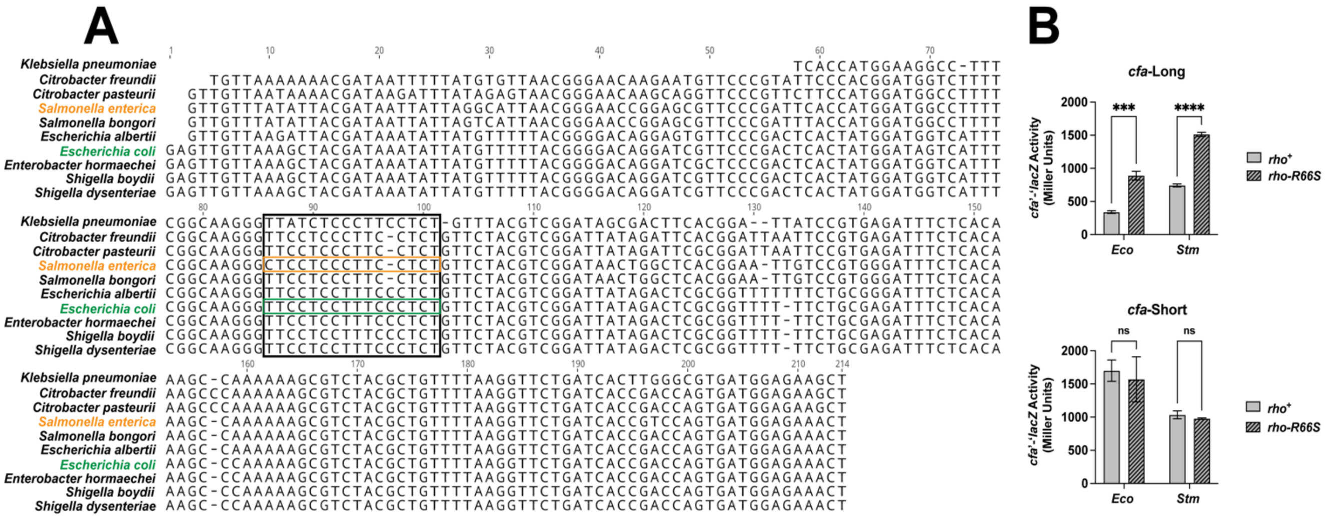

Figure S2. The long 5' UTR of *cfa* mRNA from *Salmonella enterica* is required for premature Rho-dependent transcription termination. A. Nucleotide sequence alignment of *cfa* mRNA 5' UTR sequences from ten different enterobacterial species. The larger boxed region contains the portion of each sequence aligned with the *E. coli* CU-rich region (green box). The orange box highlights the *S. enterica cfa* CU-rich sequence. The alignment was generated using the Clustal Omega program (version 1.2.3) through Geneious Prime software (version 2023.2.1). **B.** β-galactosidase activity of *E. coli* (*Eco*) and *S. enterica* (*Stm*) *cfa*-Long and *cfa*-Short fusions was measured at mid-exponential phase. Error bars represent the standard deviations of three biological replicates and statistical significance was determined using two-tailed Welch's t-tests (****P < 0.001).

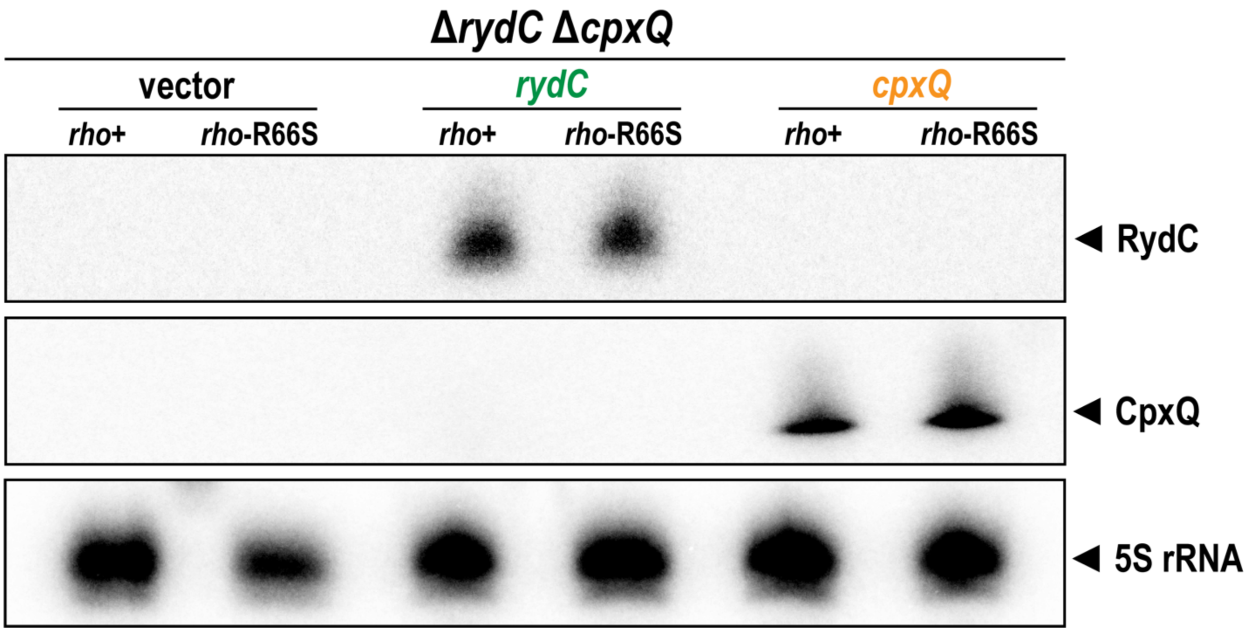

Figure S3. RydC and CpxQ are expressed at similar levels in wild-type and *rho* mutant strains. Northern blot analysis to examine the levels of RydC and CpxQ sRNAs in the presence of a vector control or RydC- or CpxQ-producing plasmids in wild-type (Δ*rydC* Δ*cpxQ rho*+) and *rho* mutant (Δ*rydC* Δ*cpxQ rho*-R66S) cells. The 5S rRNA was used as an RNA loading control.

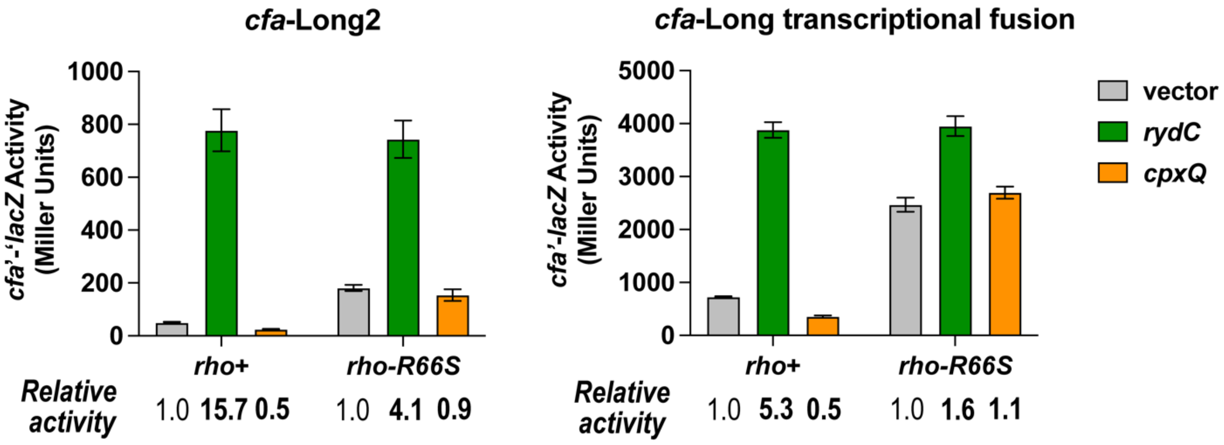

Figure S4. sRNA-dependent regulation of the *cfa*-Long2 translational fusion and *cfa*-Long transcriptional fusion. β-galactosidase activity of the *cfa*-Long2 translational fusion and the *cfa*-Long transcriptional fusion was measured at mid-exponential phase in *rho*+ and *rho*-R66S mutant backgrounds in presence of a vector control or RydC- or CpxQ-producing plasmids. Error bars represent the standard deviations of three biological replicates.

Table S1. Plasmids and strains used in this study.

**Plasmid**	**Vector**	**Genotype**	**Source or Reference**
pBRCS12	pHDB3	vector control	Wadler et al., 2009
pCB4	pBRCS12	P_{lac}-*arrS*	Bianco 2019
pCB5	pBRCS12	P_{lac}-*cpxQ*	Bianco 2019
pCB7	pBRCS12	P_{lac}-*rydC*	Bianco 2019
pCB11	pBRCS12	P_{lac}-*oxyS*	Bianco 2019
pCB12	pBRCS12	P_{lac}-*gcvB*	Bianco 2019
pAKB001	pBRCS12	P_{lac}-*cpxQ_C10G_C11G*	This study
pAKB002	pBRCS12	P_{lac}-*cpxQ_G9U*	This study
**Strain**	**Parent**	**Genotype**	**Source or Reference**
DJ480	MG1655	Δ*lac* X74	D. Jin, NCI
EM1277	DJ480 (EM1055)	Δ*lac* X74 *rne-3071* (ts) *zce-726*::Tn10	Masse, Escorcia, and Gottesman 2003
PM1805	PM1205	MG1655 *mal*::*lacI*^{Q} Δ*araBAD araC*+ *lacI*'::P_{BAD}-*cat-sacB-lacZ* miniλ-tet^{R}	Lee and Gottesman 2016
AK27	PM1205	P_{BAD}-*cfa*'-'*lacZ*-Long	King 2019
AK28	PM1205	P_{BAD}-*cfa*'-'*lacZ*-Short	King 2019
CB839	PM1205	P_{BAD}-*cfa*'-'*lacZ*-LongG3	Bianco 2019
CB1118	CB839	P_{BAD}-*cfa*'-'*lacZ*-LongG3 *rho*-R66S	This study
CB1089	DJ480	Δ*lac* X74 *rho*-R66S	This study
CB1097	AK28	P_{BAD}-*cfa*'-'*lacZ*-Short *rho*-R66S	This study
CB1098	AK27	P_{BAD}-*cfa*'-'*lacZ*-Long *rho*-R66S	This study
KF5	PM1805	P_{BAD}-*cfa*'-'*lacZ*-Long2	This study
KF6	PM1805	P_{BAD}-*cfa*'-'*lacZ*-Short2	This study
KF16	KF5	P_{BAD}-*cfa*'-'*lacZ*-Long2 *rho*-R66S	This study
KF17	KF6	P_{BAD}-*cfa*'-'*lacZ*-Short2 *rho*-R66S	This study
KF43	PM1805	P_{BAD}-*rho*'-'*lacZ*	This study
KF61	KF43	P_{BAD}-*rho*'-'*lacZ* *rho*-R66S	This study
KF88	PM1805	P_{BAD}-*cfa*'-'*lacZ*-Long G fusion	This study
KF92	PM1805	P_{BAD}-*cfa*'-'*lacZ*-Long GGG fusion	This study
KF94	PM1805	P_{BAD}-*cfa*'-'*lacZ*-Long GGGGGG fusion	This study

KF100	KF88	P_{BAD}-*cfa*'-'*lacZ*-Long G fusion *rho*-R66S	This study
KF102	KF92	P_{BAD}-*cfa*'-'*lacZ*-Long GGG fusion *rho*-R66S	This study
KF103	KF94	P_{BAD}-*cfa*'-'*lacZ*-Long GGGGGG fusion *rho*-R66S	This study
KF130	KF128	Δ*cpxQ* Δ*rydC*	This study
KF145	PM1805	P_{BAD}-*cfa*'-'*lacZ*-Long (*S. enterica*)	This study
KF147	PM1805	P_{BAD}-*cfa*'-'*lacZ*-Short (*S. enterica*)	This study
KF149	KF145	P_{BAD}-*cfa*'-'*lacZ*-Long (*S. enterica*) *rho*-R66S	This study
KF150	KF147	P_{BAD}-*cfa*'-'*lacZ*-Short (*S. enterica*) *rho*-R66S	This study
KF152	KF130	Δ*cpxQ* Δ*rydC* *rho*-R66S	This study
KF181	AK27	P_{BAD}-*cfa*'-'*lacZ*-Long *rne131*	This study
KF184	KF181	P_{BAD}-*cfa*'-'*lacZ*-Long *rne131* *rho*-R66S	This study
KF217	DJ480	Δ*lac* X74 *rne131*	This study
KF264	PM1805	P_{BAD}-*cfa*'-*lacZ*-Long	This study
KF265	PM1805	P_{BAD}-*cfa*'-*lacZ*-Short	This study
KF266	KF264	P_{BAD}-*cfa*'-*lacZ*-Long *rho*-R66S	This study
KF267	KF265	P_{BAD}-*cfa*'-*lacZ*-Short *rho*-R66S	This study
KF279	EM1277	Δ*lac* X74 *rne-3071* (ts) Cm^R *zce-726*::Tn10	This study
KF308	KF279	Δ*lac* X74 *rne-3071* (ts) Cm^R *zce-726*::Tn10 *rho*-R66S	This study
KF290	PM1805	P_{BAD}-*cfa*'-*lacZ*-Long +58	This study
KF291	PM1805	P_{BAD}-*cfa*'-*lacZ*-Long +78	This study
KF292	PM1805	P_{BAD}-*cfa*'-*lacZ*-Long +98	This study
KF293	PM1805	P_{BAD}-*cfa*'-*lacZ*-Long +118	This study
KF294	PM1805	P_{BAD}-*cfa*'-*lacZ*-Long +138	This study
KF295	PM1805	P_{BAD}-*cfa*'-*lacZ*-Long +158	This study
KF300	KF290	P_{BAD}-*cfa*'-*lacZ*-Long +58 *rho*-R66S	This study
KF301	KF291	P_{BAD}-*cfa*'-*lacZ*-Long +78 *rho*-R66S	This study
KF302	KF292	P_{BAD}-*cfa*'-*lacZ*-Long +98 *rho*-R66S	This study

KF303	KF293	P_{BAD}-*cfa*'-*lacZ*-Long +118 *rho*-R66S	This study
KF304	KF294	P_{BAD}-*cfa*'-*lacZ*-Long +138 *rho*-R66S	This study
KF305	KF295	P_{BAD}-*cfa*'-*lacZ*-Long +158 *rho*-R66S	This study
AKB078	KF264	P_{BAD}-*cfa*'-'*lacZ*-Long G78C/G79C (WS)	This study
AKB079	KF264	P_{BAD}-*cfa*'-'*lacZ*-Long G78C/G79C (WS) *rho*-R66S	This study
AKB080	KF264	P_{BAD}-*cfa*'-'*lacZ*-Long C80A (SS)	This study
AKB081	KF264	P_{BAD}-*cfa*'-'*lacZ*-Long C80A (SS) *rho*-R66S	This study

Table 2. Oligonucleotides used in this study.

Oligo	Description	Sequence (5' to 3')
cfa-Long2 Forward	Forward primer for *cfa*'-'*lacZ*-Long2 fusion construction	ACCTGACGCTTTTTATCGCAACTCTCTACTGTTT CTCCATGAGTTGTTAAAGCTACGATA
cfa-Short2 Forward	Forward primer for *cfa*'-'*lacZ*-Short2 fusion construction	ACCTGACGCTTTTTATCGCAACTCTCTACTGTTTCTC CATAAGGTTCTGATCACCGACCA
cfa-2 Reverse	Reverse primer for *cfa*'-'*lacZ*-Long2 and *cfa*'-'*lacZ*-Short2 fusion construction	TAACGCCAGGGTTTTCCCAGTCACGACGTTGTAAA ACGACGACGCCCACCACGCTTACGT
cfa-Long G mutant gBlock	gBlock gene fragment for *cfa*'-'*lacZ*-Long G fusion construction	TCGCAACTCTCTACTGTTTCTCCATGAGTTGTTAAAG CTACGATAAATATTATGTTTTTACGGGGACAGGATCG TTCCCGACTCACTATGGATAGTCATTTCGGCAAGGGT TCCTCCTTTCCCTGTGTTCTACGTCGGATTATAGACT CGCGGTTTTTTCTGCGAGATTTCTCACAAAGCCCAAA AAGCGTCTACGCTGTTTTAAGGTTCTGATCACCGACC AGTGATGGAGAAACTATGAGTTCATCGTGTATAGAA GAAGTCAGTGTACCGGATGACAACTGGTACCGTATC GCCAACGAATTACTTAGCCGTGCCGGTATAGCCATT AACGGTTCTGCCCCGGTCGTTTTACAACGTCGTGAC TGGG
cfa-Long GGG mutant gBlock	gBlock gene fragment for *cfa*'-'*lacZ*-Long GGG	TCGCAACTCTCTACTGTTTCTCCATGAGTTGTTAAAG CTACGATAAATATTATGTTTTTACGGGGACAGGATCG TTCCCGACTCACTATGGATAGTCATTTCGGCAAGGGT TCCTCCTTTGGGTCTGTTCTACGTCGGATTATAGACT

	fusion construction	CGCGGTTTTTTCTGCGAGATTTCTCACAAAGCCCAAAAAGCGTCTACGCTGTTTTAAGGTTCTGATCACCGACCAGTGATGGAGAAACTATGAGTTCATCGTGTATAGAAGAAGTCAGTGTACCGGATGACAACTGGTACCGTATCGCCAACGAATTACTTAGCCGTGCCGGTATAGCCATTAACGGTTCTGCCCCGGTCGTTTTACAACGTCGTGACTGG
cfa-Long GGGGGG mutant gBlock	gBlock gene fragment for *cfa*’-‘*lacZ*-Long GGGGGG fusion construction	TCGCAACTCTCTACTGTTTCTCCATGAGTTGTTAAAGCTACGATAAATATTATGTTTTTACGGGGACAGGATCGTTCCCGACTCACTATGGATAGTCATTTCGGCAAGGGTTCCTGGTTTGGGTGTGTTCTACGTCGGATTATAGACTCGCGGTTTTTTCTGCGAGATTTCTCACAAAGCCCAAAAAGCGTCTACGCTGTTTTAAGGTTCTGATCACCGACCAGTGATGGAGAAACTATGAGTTCATCGTGTATAGAAGAAGTCAGTGTACCGGATGACAACTGGTACCGTATCGCCAACGAATTACTTAGCCGTGCCGGTATAGCCATTAACGGTTCTGCCCCGGTCGTTTTACAACGTCGTGACTGGG
Stm cfa-Long Forward	Forward primer for *S. enterica cfa*’-‘*lacZ*-Long fusion construction	ACCTGACGCTTTTTATCGCAACTCTCTACTGTTTCTCCATCGGTTGTTTATATTACGATA
Stm cfa-Short Forward	Forward primer for *S. enterica cfa*’-‘*lacZ*-Short fusion construction	ACCTGACGCTTTTTATCGCAATCTTCTACTCTTTCTCCATAAGGTTCTGATCACCGTCCA
Stm cfa Reverse	Reverse primer for *S. enterica cfa*’-‘*lacZ*-Long and *cfa*’-‘*lacZ*-	TAACGCCAGGGTTTTCCCAGTCACGACGTTGTAAAACGACCGGGGCGGAACCATTAATTG

	Short fusion construction	
cfa-Long transcriptional fusion gBlock	gBlock gene fragment for *cfa*'-*lacZ*-Long transcriptional fusion construction	ACCTGACGCTTTTTATCGCAACTCTCTACTGTTTCTCCATGAGTTGTTAAAGCTACGATAAATATTATGTTTTTACGGGGACAGGATCGTTCCCGACTCACTATGGATAGTCATTTCGGCAAGGGTTCCTCCTTTCCCTCTGTTCTACGTCGGATTATAGACTCGCGGTTTTTTCTGCGAGATTTCTCACAAAGCCCAAAAAGCGTCTACGCTGTTTTAAGGTTCTGATCACCGACCAGTGATGGAGAAACTATGAGTTCATCGTGTATAGAAGAAGTCAGTGTACCGGATGACAACTGGTACCGTATCGCCAACGAATTACTTAGCCGTGCCGGTATAGCCATTAACGGTTCTGCCCCGATTTCACACAGGAAACAGCTATGACCATGATTACGGATTCACTGGCCGTCGTTTTACAACGTCGTGACTGGGAAAACCCTGGCGTTA
cfa-Short transcriptional fusion gBlock	gBlock gene fragment for *cfa*'-*lacZ*-Short transcriptional fusion construction	ACCTGACGCTTTTTATCGCAACTCTCTACTGTTTCTCCATAAGGTTCTGATCACCGACCAGTGATGGAGAAACTATGAGTTCATCGTGTATAGAAGAAGTCAGTGTACCGGATGACAACTGGTACCGTATCGCCAACGAATTACTTAGCCGTGCCGGTATAGCCATTAACGGTTCTGCCCCGATTTCACACAGGAAACAGCTATGACCATGATTACGGATTCACTGGCCGTCGTTTTACAACGTCGTGACTGGGAAAACCCTGGCGTTA
cfa-Long +58 transcriptional fusion gBlock	gBlock gene fragment for *cfa*'-*lacZ*-Long +58 transcriptional fusion construction	ACCTGACGCTTTTTATCGCAACTCTCTACTGTTTCTCCATGAGTTGTTAAAGCTACGATAAATATTATGTTTTTACGGGGACAGGATCGTTCCCGACTATTTCACACAGGAAACAGCTATGACCATGATTACGGATTCACTGGCCGTCGTTTTACAACGTCGTGACTGGGAAAACCCTGGCGTTA
cfa-Long +78 transcriptional fusion gBlock	gBlock gene fragment for *cfa*'-*lacZ*-Long +78 transcriptional	ACCTGACGCTTTTTATCGCAACTCTCTACTGTTTCTCCATGAGTTGTTAAAGCTACGATAAATATTATGTTTTTACGGGGACAGGATCGTTCCCGACTCACTATGGATAGTCATTTCGATTTCACACAGGAAACAGCTATGA

	fusion construction	CCATGATTACGGATTCACTGGCCGTCGTTTTACAACGTCGTGACTGGGAAAACCCTGGCGTTA
cfa-Long +98 transcriptional fusion gBlock	gBlock gene fragment for *cfa*'-*lacZ*-Long +98 transcriptional fusion construction	ACCTGACGCTTTTTATCGCAACTCTCTACTGTTTCTCCATGAGTTGTTAAAGCTACGATAAATATTATGTTTTTACGGGGACAGGATCGTTCCCGACTCACTATGGATAGTCATTTCGGCAAGGGTTCCTCCTTTCCCATTTCACACAGGAAACAGCTATGACCATGATTACGGATTCACTGGCCGTCGTTTTACAACGTCGTGACTGGGAAAACCCTGGCGTTA
cfa-Long +118 transcriptional fusion gBlock	gBlock gene fragment for *cfa*'-*lacZ*-Long +118 transcriptional fusion construction	ACCTGACGCTTTTTATCGCAACTCTCTACTGTTTCTCCATGAGTTGTTAAAGCTACGATAAATATTATGTTTTTACGGGGACAGGATCGTTCCCGACTCACTATGGATAGTCATTTCGGCAAGGGTTCCTCCTTTCCCTCTGTTCTACGTCGGATTATATTTCACACAGGAAACAGCTATGACCATGATTACGGATTCACTGGCCGTCGTTTTACAACGTCGTGACTGGGAAAACCCTGGCGTTA
cfa-Long +138 transcriptional fusion gBlock	gBlock gene fragment for *cfa*'-*lacZ*-Long +138 transcriptional fusion construction	ACCTGACGCTTTTTATCGCAACTCTCTACTGTTTCTCCATGAGTTGTTAAAGCTACGATAAATATTATGTTTTTACGGGGACAGGATCGTTCCCGACTCACTATGGATAGTCATTTCGGCAAGGGTTCCTCCTTTCCCTCTGTTCTACGTCGGATTATAGACTCGCGGTTTTTTCTGCATTTCACACAGGAAACAGCTATGACCATGATTACGGATTCACTGGCCGTCGTTTTACAACGTCGTGACTGGGAAAACCCTGGCGTTA
cfa-Long +158 transcriptional fusion gBlock	gBlock gene fragment for *cfa*'-*lacZ*-Long +158 transcriptional fusion construction	ACCTGACGCTTTTTATCGCAACTCTCTACTGTTTCTCCATGAGTTGTTAAAGCTACGATAAATATTATGTTTTTACGGGGACAGGATCGTTCCCGACTCACTATGGATAGTCATTTCGGCAAGGGTTCCTCCTTTCCCTCTGTTCTACGTCGGATTATAGACTCGCGGTTTTTTCTGCGAGATTTCTCACAAAGCCCAATTTCACACAGGAA

		ACAGCTATGACCATGATTACGGATTCACTGGCCGT CGTTTTACAACGTCGTGACTGGGAAAACCCTGGCG TTA
Fusion check Forward	Forward primer for validation of translational fusions	TCGCAACTCTCTACTGTTTCTCCAT
Fusion check Reverse	Reverse primer for validation of translational fusions	CCCAGTCACGACGTTGTAAAACGAC
rho fusion Forward	Forward primer for *rho*'-'*lacZ* fusion construction	ACCTGACGCTTTTTATCGCAACTCTCTACTGTTTCT CCATGACTTCGTATTAAACATACC
rho fusion Reverse	Reverse primer for *rho*'-'*lacZ* fusion construction	TAACGCCAGCCTTTTCCCAGTCACGACGTTCTAAAAC GACCGGCGTATTCTTTAATTCGG
rho-R66S check Forward	Forward primer to check for *rho*-R66S mutation	GCTCGTATGCGTAAGCAGGA
rho-R66S check Reverse	Reverse primer to check for *rho*-R66S mutation	GCGCAAAATAGCGTTCACCT
rne131 check Forward	Forward primer to check for *rne131* mutation	CGCGCCTGTTGTAGCTCCAG
rne131 check Reverse	Reverse primer to check for *rne131* mutation	TTCGCGATTATCGCTGCCTT

rne3071-cat linkage Forward	Forward primer to link *cat* gene to *rne3071* allele	TGGGATCGCTGGGGCGGGCATTTTTTTGCCTATTTT GCATGTGTAGGCTGGAGCTGCTTC
rne3071-cat linkage Reverse	Reverse primer to link *cat* gene to *rne3071* allele	CATATTAAAATTCATCGAATGGCATCCTTGCTAACCA ACACATATGAATATCCTCCTTAG
rne3071 check Forward	Forward primer to check for *rne3071* mutation	AACGCAACTCAGCAGGAAGA
rne3071 check Reverse	Reverse primer to check for *rne3071* mutation	AGACGAACTTCACGCTGGAA
cpxQ deletion check Forward	Forward primer to check *cpxQ* deletion	TCACGTTCCCAGTAGTAAAC
cpxQ deletion check Reverse	Reverse primer to check *cpxQ* deletion	ATGCATTAAGCAGCAGGCAA
rydC deletion check Forward	Forward primer to check *rydC* deletion	CGCGTAAACGTTCCTGAAGG
rydC deletion check Reverse	Reverse primer to check *rydC* deletion	GGACGTATGGGCAAGGATTA
cfa 5'UTR Forward	Forward primer for *cfa* 5'UTR RT-qPCR	GTTTTTACGGGGACAGGATCG
cfa 5'UTR Probe	Probe for *cfa* 5'UTR RT-qPCR	56-FAM-TCCCGACTC-ZEN-ACTATGGATAGT-3IABkFQ

cfa 5'UTR Reverse	Reverse primer for *cfa* 5'UTR RT-qPCR	GGAGGAACCCTTGCCGAAAT
cfa 5'ORF Forward	Forward primer for *cfa* 5'ORF RT-qPCR	AAGAAGGCTCTTTGGGGTTAGG
cfa 5'ORF Probe	Probe for *cfa* 5'ORF RT-qPCR	56-FAM-GATGGCTGG-ZEN-TGGGAATGTGA-3IABkFQ
cfa 5'ORF Reverse	Reverse primer for *cfa* 5'ORF RT-qPCR	AATGATGGGGGAGTTGGTTCTC
cfa 3'ORF Forward	Forward primer for *cfa* 3'ORF RT-qPCR	ACGATACCTATTTTGCGGTGGT
cfa 3'ORF Probe	Probe for *cfa* 3'ORF RT-qPCR	56-FAM-GGAAGGCAT-ZEN-ATTCCTGCTCCA-3IABkFQ
cfa 3'ORF Reverse	Reverse primer for *cfa* 3'ORF RT-qPCR	CAGGCAACCGTTCGGAAAAATA
rho 5'UTR Forward	Forward primer for *rho* 5'UTR RT-qPCR	GCTCGTCACTCAATCCGTCT
rho 5'UTR Probe	Probe for *rho* 5'UTR RT-qPCR	56-FAM-TTCTGCGTA-ZEN-CTCTCCTGTGA-3IABkFQ
rho 5'UTR Reverse	Reverse primer for *rho* 5'UTR RT-qPCR	CATGTCTTTTCGCTGCCTGG
rho 5'ORF Forward	Forward primer for *rho* 5'ORF RT-qPCR	AACCTCCGCACTGGTGATAC
rho 5'ORF Probe	Probe for *rho* 5'ORF RT-qPCR	56-FAM-TCTCTGGTA-ZEN-AGATTCGCCCG-3IABkFQ

rho 5'ORF Reverse	Reverse primer for *rho* 5'ORF RT-qPCR	CGTTAACTTTCAGCAGCGCA
rho 3'ORF Forward	Forward primer for *rho* 3'ORF RT-qPCR	GCAACATGGAACTGCACCTC
gapA control Forward	Forward primer for *gapA* control RT-qPCR	AAGTGGTTATGACTGGTCCGTC
gapA control Probe	Probe for *gapA* control RT-qPCR	56-FAM-ATATGCTGG-ZEN-CCAGGACATCG-3IABkFQ
gapA control Reverse	Reverse primer for *gapA* control RT-qPCR	CGTTGATAACTTTAGCCAGCGG
cfa 5'UTR NB probe	Northern blot probe that hybridizes to the *cfa* mRNA 5'UTR	AAATGACTATCCATAGTGAGTCGGG
cfa CDS NB probe	Northern blot probe that hybridizes to the *cfa* mRNA CDS	GACGCCCACCACGCTTACGTCATAA
CpxQ NB probe	Northern blot probe that hybridizes to the CpxQ sRNA	GGGATGGTGTCTATGGCAAGGAAAA
RydC NB probe	Northern blot probe that hybridizes to the RydC sRNA	CGAAGAATACGGGTCTACATCGGAA
5S rRNA NB probe	Control northern blot probe that	GTTTCACTTCTGAGTTCGGCATGGGGTCAGGTGGG

	hybridizes to the 5S rRNA	
SsrA NB probe	Control northern blot probe that hybridizes to the SsrA tmRNA	ATCCCGTCGAATCCAGAATCAGCCC
cfa-Long WS mutant gBlock	gBlock gene fragment for *cfa*'-'*lacZ*-Long WS fusion construction	CTGACGCTTTTTATCGCAACTCTCTACTGTTTCTCCATGAGTTGTTAAAGCTACGATAAATATTATGTTTTTACGGGGACAGGATCGTTCCCGACTCACTATGGATAGTCATTTCCCCAAGGGTTCCTCCTTTCCCTCTGTTCTACGTCGGATTATAGACTCGCGGTTTTTTCTGCGAGATTTCTCACAAAGCCCAAAAAGCGTCTACGCTGTTTTAAGGTTCTGATCACCGACCAGTGATGGAGAAACTATGAGTTCATCGTGTATAGAAGAAGTCAGTGTACCGGATGACAACTGGTACCGTATCGCCAACGAATTACTTAGCCGTGCCGGTATAGCCATTAACGGTTCTGCCCCGGTCGTTTTACAACGTCGTGACTGGGAAAACCCTGGCGTTA
cfa-Long SS mutant gBlock	gBlock gene fragment for *cfa*'-'*lacZ*-Long WS fusion construction	CTGACGCTTTTTATCGCAACTCTCTACTGTTTCTCCATGAGTTGTTAAAGCTACGATAAATATTATGTTTTTACGGGGACAGGATCGTTCCCGACTCACTATGGATAGTCATTTCGGAAAGGGTTCCTCCTTTCCCTCTGTTCTACGTCGGATTATAGACTCGCGGTTTTTTCTGCGAGATTTCTCACAAAGCCCAAAAAGCGTCTACGCTGTTTTAAGGTTCTGATCACCGACCAGTGATGGAGAAACTATGAGTTCATCGTGTATAGAAGAAGTCAGTGTACCGGATGACAACTGGTACCGTATCGCCAACGAATTACTTAGCCGTGCCGGTATAGCCATTAACGGTTCTGCCCCGGTCGTTTTACAACGTCGTGACTGGGAAAACCCTGGCGTTA
CpxQ_C10G C11G Forward	Oligo for site-directed mutagenesis of pCpxQ for WS	GATCCAGTATCTTGTTATCC

	compensatory mutation	
CpxQ_C10G C11G Reverse	Oligo for site-directed mutagenesis of pCpxQ for WS compensatory mutation	CTTTTCCTTGggATAGACACCATC
CpxQ_G9U Forward	Oligo for site-directed mutagenesis of pCpxQ for SS compensatory mutation	ATCCAGTATCTTGTTATCC
CpxQ_G9U Reverse	Oligo for site-directed mutagenesis of pCpxQ for SS compensatory mutation	CCTTTTCCTTtCCATAGACAC
